# Simulated Cost-effectiveness and Long-term Clinical Outcomes of Addiction Care and Antibiotic Therapy Strategies for Patients With Injection Drug Use–Associated Infective Endocarditis

**DOI:** 10.1001/jamanetworkopen.2022.0541

**Published:** 2022-02-28

**Authors:** Joëlla W. Adams, Alexandra Savinkina, James C. Hudspeth, Mam Jarra Gai, Raagini Jawa, Laura R. Marks, Benjamin P. Linas, Alison Hill, Jason Flood, Simeon Kimmel, Joshua A. Barocas

**Affiliations:** 1 Section of Infectious Diseases, Boston Medical Center, Boston, Massachusetts; 2 RTI International, Research Triangle Park, North Carolina; 3 Department of Medicine, Boston University School of Medicine, Boston, Massachusetts; 4 Division of Infectious Diseases, School of Medicine, Washington University in St Louis, St Louis, Missouri; 5 Population Health Analytics Division, Boston Medical Center, Boston, Massachusetts; 6 Section of General Medicine, Boston Medical Center, Boston, Massachusetts; 7 Division of General Internal Medicine, Anschutz Medical Campus, University of Colorado, Aurora; 8 Division of Infectious Diseases, Anschutz Medical Campus, University of Colorado, Aurora

## Abstract

**Importance:**

Emerging evidence supports the use of outpatient parenteral antimicrobial therapy (OPAT) and, in many cases, partial oral antibiotic therapy for the treatment of injection drug use–associated infective endocarditis (IDU-IE); however, long-term outcomes and cost-effectiveness remain unknown.

**Objective:**

To compare the added value of inpatient addiction care services and the cost-effectiveness and clinical outcomes of alternative antibiotic treatment strategies for patients with IDU-IE.

**Design, Setting, and Participants:**

This decision analytical modeling study used a validated microsimulation model to compare antibiotic treatment strategies for patients with IDU-IE. Model inputs were derived from clinical trials and observational cohort studies. The model included all patients with injection opioid drug use (N = 5 million) in the US who were eligible to receive OPAT either in the home or at a postacute care facility. Costs were annually discounted at 3%. Cost-effectiveness was evaluated from a health care sector perspective over a lifetime starting in 2020. Probabilistic sensitivity, scenario, and threshold analyses were performed to address uncertainty.

**Interventions:**

The model simulated 4 treatment strategies: (1) 4 to 6 weeks of inpatient intravenous (IV) antibiotic therapy along with opioid detoxification (usual care strategy), (2) 4 to 6 weeks of inpatient IV antibiotic therapy along with inpatient addiction care services that offered medication for opioid use disorder (usual care/addiction care strategy), (3) 3 weeks of inpatient IV antibiotic therapy along with addiction care services followed by OPAT (OPAT strategy), and (4) 3 weeks of inpatient IV antibiotic therapy along with addiction care services followed by partial oral antibiotic therapy (partial oral antibiotic strategy).

**Main Outcomes and Measures:**

Mean percentage of patients completing treatment for IDU-IE, deaths associated with IDU-IE, life expectancy (measured in life-years [LYs]), mean cost per person, and incremental cost-effectiveness ratios (ICERs).

**Results:**

All modeled scenarios were initialized with 5 million individuals (mean age, 42 years; range, 18-64 years; 70% male) who had a history of injection opioid drug use. The usual care strategy resulted in 18.63 LYs at a cost of $416 570 per person, with 77.6% of hospitalized patients completing treatment. Life expectancy was extended by each alternative strategy. The partial oral antibiotic strategy yielded the highest treatment completion rate (80.3%) compared with the OPAT strategy (78.8%) and the usual care/addiction care strategy (77.6%). The OPAT strategy was the least expensive at $412 150 per person. Compared with the OPAT strategy, the partial oral antibiotic strategy had an ICER of $163 370 per LY. Increasing IDU-IE treatment uptake and decreasing treatment discontinuation made the partial oral antibiotic strategy more cost-effective compared with the OPAT strategy. When assuming that all patients with IDU-IE were eligible to receive partial oral antibiotic therapy, the strategy was cost-saving and resulted in 0.0247 additional discounted LYs. When treatment discontinuation was decreased from 3.30% to 2.65% per week, the partial oral antibiotic strategy was cost-effective compared with OPAT at the $100 000 per LY threshold.

**Conclusions and Relevance:**

In this decision analytical modeling study, incorporation of OPAT or partial oral antibiotic approaches along with addiction care services for the treatment of patients with IDU-IE was associated with increases in the number of people completing treatment, decreases in mortality, and savings in cost compared with the usual care strategy of providing inpatient IV antibiotic therapy alone.

## Key Points

**Question:**

What is the most clinically beneficial and cost-effective antibiotic treatment strategy for injection drug use–associated infective endocarditis (IDU-IE)?

**Findings:**

In this decision analytical modeling study simulating 4 treatment strategies among 5 million individuals with IDU-IE in the US, a validated microsimulation model suggested that outpatient parenteral antimicrobial therapy was the most cost-effective strategy for the treatment of IDU-IE. A partial oral antibiotic treatment strategy was associated with the highest treatment completion rate and was most cost-effective when methicillin-resistant *Staphylococcus aureus* was not a causative pathogen.

**Meaning:**

This study found that outpatient parenteral antimicrobial therapy and partial oral antibiotic therapy regimens were likely to be as clinically beneficial as and less costly than 6 weeks of inpatient intravenous antibiotic therapy for the treatment of IDU-IE.

## Teaser

This decision analytical modeling study assesses the added value of inpatient addiction care services and compares the cost-effectiveness and clinical outcomes of different antibiotic treatment strategies among 5 million individuals with injection drug use–associated infective endocarditis in the US.

## Introduction

Hospitalizations associated with infective endocarditis in the US increased from 16 per 100 000 adults in 2003 to 22 per 100 000 adults in 2016.^1^ Injection drug use–associated infective endocarditis (IDU-IE) currently accounts for 1 in 10 hospitalizations for infective endocarditis.^2^ This increase has largely been associated with the opioid epidemic, specifically the injection of heroin and fentanyl. If current patterns continue, more than 250 000 individuals in the US may die of IDU-IE between 2020 and 2030.^3^ There is a substantial need to define optimal treatment strategies given the increasing burden of IDU-IE in the US.

Standard treatment for IDU-IE comprises 4 to 6 weeks of intravenous (IV) antibiotic therapy.^4^ Emerging evidence exists for the use of oral antibiotic therapy for the treatment of many types of infective endocarditis and the use of outpatient parenteral antimicrobial therapy (OPAT) for at least part of the treatment course.^5,6,7,8,9^ However, patients with IDU-IE are often required to remain hospitalized until treatment completion.^10^ Almost 20% of patients admitted with IDU-IE have a patient-directed discharge (ie, leave the hospital against medical advice).^11,12^ Alternative antibiotic treatment strategies that shorten hospitalization and allow patients to complete treatment elsewhere could increase the likelihood of treatment completion and decrease costs.

Current US treatment guidelines state that partial oral antibiotic therapy may be a reasonable option for patients with IDU-IE associated with uncomplicated right-sided *Staphylococcus aureus* infection but recommend that this approach only be used when parenteral antibiotic therapy is problematic.^13,14^ However, a retrospective cohort study^5^ found that people with IDU-IE who received a complete course of IV antibiotic therapy had similar readmission rates to those who could not complete inpatient IV antibiotic therapy and were provided partial oral antibiotic therapy at the time of patient-directed discharge.

Another strategy, OPAT, is widely used to treat infections that require prolonged antibiotic therapy, and this treatment strategy has a proven safety record.^15^ However, clinicians’ concerns regarding the misuse of a peripherally inserted central catheter to inject drugs in addition to treatment nonadherence, unstable living situations, and stigma associated with substance use have typically excluded people who inject drugs from receiving OPAT.^16,17^ Despite these concerns, a 2018 systematic review^8^ found that OPAT may be safe and beneficial for treating IDU-IE. To our knowledge, no study to date has compared the long-term impact and cost-effectiveness of OPAT with IV and partial oral antibiotic treatment strategies.

Recent research^18^ has highlighted the role of addiction care services in improving outcomes among individuals hospitalized with IDU-IE. Addiction care services, which can include addiction counseling, opioid withdrawal management, long-term medication titration, and referral and linkage to outpatient addiction care, have been reported to increase the likelihood of receiving medication for opioid use disorder (MOUD) during and after treatment for IDU-IE and have been associated with reductions in mortality risk^19^ and decreases in the probability of reinfection.^20,21^ Despite these benefits, an analysis from 1 hospital found that fewer than 8% of patients admitted with IDU-IE were discharged with any plans to start MOUD,^22^ reflective of a gap in treatment which has since been confirmed in broader studies.^19,20^

We evaluated the likely long-term clinical outcomes and cost-effectiveness of different strategies for the treatment of IDU-IE. Given the emerging evidence and unanswered questions, we aimed to (1) compare the potential value of alternative antibiotic treatment strategies and (2) estimate the impact of addiction care services among patients with IDU-IE.

## Methods

The study was approved by the Boston University Medical Campus Institutional Review Board, which reviewed the Reducing Infection Related to Drug Use Cost-Effectiveness (REDUCE) model used in the study and provided a waiver of informed consent because the study did not involve human participants. This study followed the Consolidated Health Economic Evaluation Reporting Standards (CHEERS) reporting guideline for economic evaluations of health interventions (eMethods 1 in the Supplement).^23^

### Analytic Overview

We used the REDUCE model, a validated Monte Carlo microsimulation model that simulated the natural history of injection opioid use, to compare the following treatment strategies for IDU-IE: (1) 4 to 6 weeks of inpatient IV antibiotic therapy along with opioid detoxification (usual care strategy), (2) 4 to 6 weeks of inpatient IV antibiotic therapy along with inpatient addiction care services that offered MOUD (usual care/addiction care strategy), (3) 3 weeks of inpatient IV antibiotic therapy along with addiction care services followed by OPAT (OPAT strategy), and 4) 3 weeks of IV antibiotic therapy along with addiction care services followed by partial oral antibiotic therapy (partial oral antibiotic strategy).

In 3 of the strategies (usual care/addiction care, OPAT, and partial oral antibiotic therapy), patients with IDU-IE could receive addiction care services while hospitalized. These strategies were based on the assumption that when addiction care services were implemented, hospitalized individuals would have an increased probability of receiving MOUD in addition to addiction counseling, opioid withdrawal management, long-term medication titration, and referral and linkage to outpatient addiction care at the end of hospitalization.

In the OPAT strategy, all hospitalized patients with IDU-IE transitioned to either home-based or outpatient OPAT after 3 weeks of hospitalization and the offer of addiction care services. We assumed that 50% of patients would have home infusion therapy and 50% would receive OPAT at a postacute care facility. This assumption was informed by unpublished data from Boston Medical Center (A. Hill, BA, email communication, June 3, 2021) suggesting that one-half of patients with IDU-IE were homeless and therefore could not be discharged home. For the partial oral antibiotic strategy, we assumed that only patients admitted with non–methicillin-resistant *S aureus* (non-MRSA) IDU-IE would be eligible to receive oral antibiotic therapy after 3 weeks of hospitalization; however, all patients would be eligible to receive addiction care services. The probability of treatment completion and costs differed for each strategy. Details of these parameters and parameter sources are available in eMethods 1 in the Supplement.

The REDUCE model simulated a closed cohort of people who injected short-acting opioid drugs. For this analysis, we simulated a cohort over a lifetime to estimate long-term outcomes, including the mean percentage of patients completing treatment for IDU-IE, deaths associated with IDU-IE, life expectancy (measured in life-years [LYs]), mean cost per person, and incremental cost-effectiveness ratios (ICERs). We compared costs using a payer system perspective and denominated currency in 2020 US dollars. We discounted all costs and benefits by 3% annually and expressed ICERs as cost per LY gained, with a willingness-to-pay threshold of $100 000 per LY.^24^ We evaluated LYs rather than quality-adjusted LYs because the interventions focused on mortality-based outcomes, and quality-adjusted LYs are intended to measure life expectancy among patients with diseases in which there is a measurable change in quality of life (eg, heart failure) and the experience with drug use is heterogeneous. Probabilistic sensitivity, scenario, and threshold analyses were performed to evaluate major findings.

### REDUCE Model Overview

#### Model Structure and Simulated Cohort

The REDUCE model has been described in detail elsewhere.^3^ The model simulated a closed cohort experiencing the natural history of injection opioid drug use. Individuals moved through time in weekly steps from model initialization until death. Each week, there was a probability of developing sequelae of injection opioid drug use (eg, overdose or IDU-IE), requiring hospitalization, receiving outpatient addiction care, and changing injection drug use behavior.

The simulated cohort was stratified by sex (male or female), age (0-99 years), and injection behavior profile, which included injection frequency (high, low, or not currently injecting drugs), sharing of injection equipment (yes, no, or never), and sterile injection technique (cleaning, no cleaning, or never) (eMethods 1 and eTable 1 in the Supplement).

#### Sequelae of Drug Use

We assumed that individuals with high-frequency injection drug use had a higher probability of both overdose and IDU-IE, and individuals who shared injection equipment or used unsterile injection techniques had a higher probability of IDU-IE (eMethods 1, eTable 2, and eTable 3 in the Supplement). Overdose and infection risks were stratified by age and sex.

#### Hospitalization

We assumed that after developing IDU-IE or experiencing an overdose, individuals had a probability of hospitalization. While hospitalized, patients could receive MOUD and a consultation for addiction care services, if available. We also assumed that individuals receiving MOUD and addiction care services had a higher probability of linking to outpatient MOUD and addiction care, and both outcomes changed the probability of decreasing the frequency of injection drug use (eMethods 2 and eTable 4 in the Supplement). Not all patients receiving addiction care services began receiving MOUD. While hospitalized, patients had a probability of leaving before treatment completion. We assumed that patients who did not complete treatment for IDU-IE would remain infected until they were readmitted to the hospital or died.

#### Outpatient Treatment Services

We assumed that when individuals left the hospital, they had a probability of linking to outpatient addiction care services and MOUD (eMethods 2 and eTable 5 in the Supplement). Linkage could be increased through receipt of inpatient addiction care services and MOUD but could also occur spontaneously through a background mechanism reflecting outpatient addiction care uptake in the nonhospitalized population.

The OPAT and partial oral antibiotic strategies simulated the provision of outpatient antibiotic therapy to individuals with IDU-IE (eMethods 3 and eTables 6-12 in the Supplement). We incorporated a weekly probability of discontinuing treatment for IDU-IE.

#### Mortality

We assumed that individuals had a probability of dying of overdose and IDU-IE in addition to age-, sex-, and drug use–adjusted mortality from competing causes of death (eMethods 2 in the Supplement). At hospitalization, individuals had an additional mortality risk applied to reflect inpatient mortality.

#### Costs

Each patient accrued costs associated with opioid use, hospitalization, and outpatient services. Care costs associated with opioid use varied by injection behavior profile. The cost analysis also accounted for health care services, stratified by age and sex, that were not associated with opioid use based on data from the Medical Expenditure Panel Survey.^25^

### Model Data and Parameter Estimation

Population, sequelae of drug use, inpatient, outpatient, mortality, and cost parameters were included in the model. The parameters and data sources are summarized in Table 1,^4,5,6,7,8,11,25,26,27,28,29,30,31,32,33,34,35,36,37,38,39,40,41,42,43,44,45,46,47,48,49,50,51,52,53,54,55,56,57,58,59,60,61,62,63,64^ and full details are available in eTable 1 to eTable 12 in the Supplement.

**Table 1.** Estimates for Important Model Parameters to Characterize Outcomes of People Who Inject Drugs Over a Lifetime

Parameter^a^	Estimate	Range	Source
**Population**			
Probability of ever drug use	100% of cohort ever injected drugs; age and sex mix informed by literature	NA	Lansky et al,^26^ 2014; Martins et al,^27^ 2017; Degenhardt et al,^28^ 2017; CDC,^29^ 2021; US Census Bureau,^30^ 2018
Probability of injection drug use frequency	Varied by age and sex	NA	Tan et al,^31^ 2018; Buresh et al,^32^ 2019
**Sequelae of drug use**			
Probability of overdose			
Low-frequency injection drug use	0.0026	0.0026-0.0027	CDC,^29^ 2021; Hser et al,^33^ 2017; Hudgins et al,^34^ 1995; Cedarbaum & Banta-Green,^35^ 2016; MDPH,^36^ 2017; MDPH,^37^ 2020; Hedegaard et al,^38^ 2018
High-frequency injection drug use	0.0005	0.0005-0.0006	
Probability of fatal overdose	0.1300	0.1200-0.2400	MDPH,^36^ 2017; MDPH,^37^ 2020; Hedegaard et al,^38^ 2018
Proportion of IDU-IE infections	100	NA	Assumed
Probability of linking to inpatient care after nonfatal overdose	0.9700	NA	Expert opinion^b^
Probability of linking to inpatient care for IDU-IE	0.2000	0.1830-0.2170	N’Guyen et al,^39^ 2017
Probability of linking to inpatient care for SSTI	0.0019	0.0008-0.0040	Hope et al,^40^ 2015
Previous overdose multiplier for risk of subsequent overdose, No. of nonfatal overdoses			
1	1.15	0.72-1.82	Caudarella et al,^41^ 2016
2-3	1.81	1.19-2.27	
4-7	2.12	1.11-4.04	
≥8	5.24	1.56-17.01	
Previous infection multiplier for risk of subsequent infection	2.80	1.50-5.10	Alagna et al,^42^ 2014
**Inpatient**			
Duration of hospitalization with IDU-IE using usual care scenarios, mean, wk	6	4-8	Miller and Polgreen,^4^ 2019
Probability of patient-directed discharge	0.0500	0.0300-0.1000	Kimmel et al,^11^ 2021; Meisner et al,^43^ 2020
Probability of addiction consultation service uptake, if available	0.2580	0.0400-0.4000	Unpublished BMC addiction care data; expert communication^c^
Probability of initiation of MOUD with an addiction consultation	0.6500	0.3200-0.9700	Unpublished ALIVE data; Priest et al,^44^ 2020; Murphy et al,^45^ 2019; Englander et al,^46^ 2020^d^
Probability of initiation of MOUD without an addiction consultation	0.1100	0.0500-0.1600	
Probability of initiation of OPAT	0.5360	0.159-0.587	Expert opinion
Probability of initiation of POA therapy	0.2290	0.159-0.3188	Rodger et al,^47^ 2018
**Outpatient**			
Antibiotic treatment			
Duration of OPAT, wk	3	2-4	Fanucchi et al,^6^ 2020
Duration of POA therapy, wk	3	2-4	Marks et al,^5^ 2020
Probability of discontinuing OPAT	0.0454	0.0300-0.1400	Fanucchi et al,^6^ 2020; D’Couto et al,^7^ 2018; Suzuki et al,^8^ 2018
Probability of discontinuing POA therapy	0.0330	0.0200-0.1100	Marks et al,^5^ 2020
Addiction care and MOUD linkage			
Link to outpatient addiction care with MOUD after inpatient addiction care with MOUD	0.7000	0.6700-0.7220	Unpublished data; Liebschutz et al,^48^ 2014; Trowbridge et al,^49^ 2017^e^
Link to outpatient addiction care with MOUD after inpatient MOUD without addiction care	0.5714	0.5404-0.6024	Unpublished data^e^
Link to outpatient addiction care without MOUD after inpatient addiction care without MOUD	0.4529	0.4415-0.4643	Unpublished data^e^
Link to outpatient addiction care without MOUD after inpatient MOUD without addiction care	0.0500	0.0490-0.0501	Knudsen et al,^50^ 2011; Larochelle et al,^51^ 2018
MOUD initiation			
Link to outpatient addiction care after inpatient addiction care	0.5069	0.4649-0.5489	Unpublished data^e^
Link to outpatient addiction care after no inpatient addiction care	0.1620	0.1439-0.3430	Knudsen et al,^50^ 2011
Unlinkage			
Spontaneous unlinking from outpatient addiction care and MOUD	0.0481	0.0298-0.0666	Liebschutz et al,^48^ 2014; Morgan et al,^52^ 2018
Spontaneous unlinking from outpatient addiction care and no MOUD	0.1560	0.1262-0.1860	Liebschutz et al,^48^ 2014; Wakeman et al,^53^ 2017
**Mortality**			
Background overdose–subtracted mortality	Varied by age and sex	0.0008-0.0011	Chang et al,^54^ 2017; Arias,^55^ 2012
Probability of death			
Untreated IDU-IE	0.1623	0.0848-0.5358	Verhagen et al,^56^ 2006; Veldhuizen and Callaghan,^57^ 2014
Untreated SSTI	0.0023	0.0023-0.0028	Veldhuizen and Callaghan,^57^ 2014
Inpatient with IDU-IE	0.0100	0.0018-0.0161	Veldhuizen and Callaghan,^57^ 2014; Rodger et al,^47^ 2018; Cresti et al,^58^ 2017; Hill et al,^59^ 2007; Ternhag et al,^60^ 2013
Inpatient with SSTI	0.0008	0.0008-0.0025	Veldhuizen and Callaghan,^57^ 2014
Inpatient with overdose	0.0190	0.0130-0.0270	Jiang et al,^61^ 2017
**Costs, $**			
Background costs	Varied by age and sex	NA	AHRQ,^25^ 2021
Frequency of injection drug use			
No current use	224	112-336	Murphy et al,^45^ 2019
High	357	178-536	Behrends et al,^62^ 2019
Low	238	119-357	Murphy et al,^45^ 2019
Overdose			
Fatal	430	215-645	Behrends et al,^62^ 2019
Nonfatal without hospitalization	1118	559-1678	Behrends et al,^62^ 2019
Hospitalization			
With IDU-IE	21 573	8736-34 410	Miller and Polgreen,^4^ 2019
With SSTI	17 751	9124-26 378	Miller and Polgreen,^4^ 2019
With overdose	14 195	12 744-15 646	Behrends et al,^62^ 2019
Addiction care services	225	150-300	Unpublished BMC addiction care data; CMS,^63^ 2020^f^
POA medications and services	380	137-1289	CMS,^63^ 2020; CMS,^64^ 2020
Outpatient			
OPAT at postacute care facility	2702	762-11 756	Unpublished BMC data^g^
Home-based OPAT medications and services	469	461-479	CMS,^63^ 2020; CMS,^64^ 2020
Addiction consultation with MOUD	81	78-138	CMS,^63^ 2020; CMS,^64^ 2020
Addiction consultation without MOUD	81	62-138	Murphy et al,^45^ 2019; CMS,^63^ 2020; CMS,^64^ 2020

Abbreviations: AHRQ, Agency for Healthcare Research and Quality; ALIVE, AIDS Linked to the Intravenous Experience study; BMC, Boston Medical Center; CDC, Centers for Disease Control and Prevention; CMS, Centers for Medicare & Medicaid Services; IDU-IE, injection drug use–associated infective endocarditis; MDPH, Massachusetts Department of Health; MOUD, medication for opioid use disorder; NA, not applicable; OPAT, outpatient parenteral antibiotic therapy; POA, partial oral antibiotic; SSTI, skin and soft tissue infection.

^a^ The REDUCE model was performed using a weekly time cycle; therefore, all probabilities are weekly.

^b^ Consensus obtained between B.P.L. and J.A.B.

^c^ Expert communication with H. Englander, MD, and C. King, PhD, via email on October 20, 2019.

^d^ Unpublished ALIVE data provided by G. Kirk, MD, and S. Mehta, MD, via email communication on March 7, 2019.

^e^ Unpublished data provided by K. Priest, MD, via email communication on October 20, 2019.

^f^ Unpublished BMC data provided by Z.M. Weinstein, MD, via email communication on March 12, 2019.

^g^ Unpublished BMC data provided by A. Hill, BA, via email communication on June 3, 2021.

#### Overdose and Hospitalization

We derived rates of fatal and nonfatal overdose from state-level data.^36,37,38^ Rates of IDU-IE were derived from the published literature.^4,65,66,67^

Data from the published literature and expert opinion (H. Englander, MD, and C. King, PhD, email communication, October 20, 2019) informed the rates of hospitalization, the probability of initiating MOUD while an inpatient, and the association of addiction care services and MOUD with injection frequency. We assumed that 26% of individuals accepted addiction care services while inpatients based on unpublished data from Boston Medical Center addiction care services (Z.M. Weinstein, MD, email communication, March 12, 2019).

#### Outpatient Treatment Services

We assumed that individuals receiving inpatient addiction care services and MOUD had a 70% probability of linking to outpatient MOUD compared with individuals receiving inpatient MOUD alone (45% linkage), individuals receiving addiction care services alone (57% linkage), and individuals not receiving either inpatient MOUD or addiction care services (5% linkage). We estimated the conditional probabilities of linking to outpatient MOUD based on data from cohort studies and clinical trials.^48,49^ The probabilities of completing OPAT and partial oral antibiotic therapies were informed by data from the published literature.^5,6,7,8^

#### Mortality and Costs

After accounting for fatal overdose, we derived age- and sex-adjusted mortality rates from the National Vital Statistics System to inform mortality associated with competing risks.^54,55^ To account for additional opioid drug use–associated harms not captured by fatal overdose or IDU-IE, we multiplied the resulting mortality rates by 1.2.^54^

We derived some of the costs from the 2020 Laboratory and Physician Fee Schedules from the Centers for Medicare and Medicaid Services^63,64^ and the Medical Expenditure Panel Survey^25^ (eTable 10 and eTable 11 in the Supplement).

#### Probabilistic, Scenario, and Threshold Analyses

For the main analysis, we performed probabilistic sensitivity analyses (eMethods 4 in the Supplement) using distributions around important model parameters. We performed 1000 simulations with 5 million individuals over a lifetime.

Deterministic sensitivity analyses were conducted to evaluate the extent of uncertainty in the input parameters (eMethods 4 in the Supplement). These analyses were performed with 500 000 individuals over a lifetime. We varied (1) the percentage of patients with IDU-IE who were eligible to receive partial oral antibiotic therapy (to reflect differences in the percentage of non-MRSA IDU-IE cases), (2) the percentage of patients leaving the hospital with patient-directed discharge, (3) the treatment uptake of OPAT and partial oral antibiotic therapy, (4) the rate of overdose within the community and outpatient settings, (5) the uptake of addiction care services and MOUD during hospitalization, and (6) the length of inpatient stay and uptake of partial oral antibiotic therapy. We also conducted threshold analyses to assess which values for selected parameters (eg, treatment uptake or treatment completion) changed our major findings (eMethods 4, eTable 13 in the Supplement).

### Statistical Analysis

The model was constructed using C++ programming language, and analyses were performed using R software, version 3.2.2 (R Foundation for Statistical Computing), and Excel software (Microsoft Corporation). No significance tests were performed for this simulation study.

## Results

We initialized the model with a cohort of 5 million individuals who reflected the age and sex of the US population who inject opioid drugs, with data informed by the US Census and published literature.^26,27,28,30,31,32,68^ At model initialization, the mean age of the cohort was 42 years (range, 18-64 years), 70% were male, 53% had high-frequency injection drug use, 11% had low-frequency injection drug use, and 36% had no current injection drug use.^26,27,28,30,31,32,68^ We assumed imperfect access to harm reduction services, with 66% of the cohort practicing unsterile injection techniques and 45% sharing injection equipment.^69^

Over a lifetime horizon within the usual care strategy, 685 637 individuals developed IDU-IE, 557 386 were hospitalized with IDU-IE, and 250 654 died of IDU-IE. The usual care strategy resulted in 18.63 LYs; 77.6% of hospitalized patients with IDU-IE completed treatment, and 5.01% of deaths in the population attributable to IDU-IE (Table 2). Life expectancy was extended by each alternative strategy (0.016 years with the usual care/addiction care strategy, 0.013 years with the OPAT strategy, and 0.024 years with the partial oral antibiotic strategy). The partial oral antibiotic strategy provided the highest treatment completion rate (80.3%) compared with the OPAT strategy (78.8%) and the usual care/addiction care strategy (77.6%). All strategies were attributable to a lower percentage of IDU-IE–associated deaths compared with the usual care strategy (4.86% with the usual care/addiction care strategy, 4.89% with the OPAT strategy, and 4.79% with the partial oral antibiotic strategy vs 5.01% with the usual care strategy) and overdose (15.70% with the usual care/addiction care strategy, 15.71% with the OPAT strategy, and 15.71% with the partial oral antibiotic strategy vs 15.73% with the usual care strategy).

**Table 2.** Selected Cost and Clinical Outcomes from Base Case Analysis^a^

Treatment strategy^b^	IDU-IE cases, No.	IDU-IE completed treatments, No. (%)	Deaths associated with IDU-IE, No. (%)	Life expectancy, y	Discounted cost, mean (95% CrI), $	Incremental discounted cost, mean, $	Hospital cost, mean, $	Discounted LY, mean (95% CrI)	Incremental discounted LY	ICER, $ per LY^c^
Usual care	685 637	432 720 (77.6)	250 654 (5.01)	73.31	416 570 (334 000-482 780)	NA	13 968	18.63 (17.28-18.67)	NA	NA
OPAT	684 867	437 547 (78.8)	244 658 (4.89)	73.34	412 150 (331 540-481 460)	4385	5450	18.65 (17.32-18.70)	0.0132	Cost-saving
POA	686 219	444 159 (80.3)	239 507 (4.79)	73.37	413 920 (333 220-483 000)	1740	8520	18.66 (17.34-18.74)	0.0106	163 370
Usual care/addiction care	684 036	438 588 (77.6)	243 176 (4.86)	73.35	416 990 (334 580-483 530)	3098	14 162	18.65 (17.30-18.70)	Dominated^d^	Dominated^d^

Abbreviations: CrI, credible interval; ICER, incremental cost-effectiveness ratio; IDU-IE, injection drug use–associated infective endocarditis; LY, life-year; NA, not applicable; OPAT, outpatient parenteral antimicrobial therapy; POA, partial oral antibiotic.

^a^ Analysis assumed that 21% of IDU-IE cases were associated with methicillin-resistant *Staphylococcus aureus* and ineligible for POA therapy; 95% CrIs were calculated, if applicable.

^b^ The usual care strategy comprised 4 to 6 weeks of inpatient intravenous (IV) antibiotic therapy along with opioid detoxification. The usual care/addiction care strategy comprised 4 to 6 weeks of inpatient IV antibiotic therapy along with inpatient addiction care services that offered MOUD. The OPAT strategy comprised 3 weeks of inpatient IV antibiotic therapy along with addiction care services followed by OPAT. The POA strategy comprised 3 weeks of inpatient IV antibiotic therapy along with addiction care services followed by POA therapy.

^c^ The overall incremental cost-effectiveness ratio was calculated as the difference in the mean discounted costs for the total US population divided the difference in the discounted quality-adjusted life expectancy for the total US population, all of which were discounted at 3% per year.

^d^ Cost more and had worse clinical outcomes.

The usual care strategy yielded a discounted lifetime mean cost of $416 570 per person (95% credible interval [CrI], $334 000-$482 780) whereas the OPAT strategy was the least expensive at $412 150 per person (95% CrI, $331 540-481 460) compared with the partial oral antibiotic strategy ($413 920 per person; 95% CrI, $333 220-$483 000) and the usual care/addiction care strategy ($416 990 per person; 95% CrI, $334 580-483 530). The usual care strategy was dominated by (ie, cost more and had worse clinical outcomes) all other strategies. Compared with the OPAT strategy, the partial oral antibiotic strategy had an ICER of $163 370 per LY. The usual care/addiction care strategy was dominated by the partial oral antibiotic strategy.

In the scenario analyses, the partial oral antibiotic strategy was preferred (ie, performed best) when patients with MRSA-associated IDU-IE were assumed to be eligible to receive partial oral antibiotic therapy, when treatment uptake of partial oral antibiotic therapy or OPAT was held equal, and when the inpatient stay was decreased and treatment uptake of partial oral antibiotic therapy was increased (Table 3). Incremental discounted LYs gained with the partial oral antibiotic strategy ranged from 0.020 (treatment uptake equal to OPAT treatment uptake) to 0.025 (MRSA-associated IDU-IE eligible for treatment), and incremental discounted costs ranged from −$4450 to −$1250. In a scenario analysis that assumed addiction care services reduced patient-directed discharge from 5.0% to 2.5% per week, the OPAT strategy was the most cost-effective, with a gain of 0.250 LYs and incremental mean discounted cost of −$4073. Increasing the uptake of addiction care services and MOUD from 25% to 75% yielded greater cost for each strategy but similar conclusions (mean discounted costs increased from $412 150 to $412 420 for the partial oral antibiotic strategy and from $413 920 to $414 300 for the OPAT strategy).

**Table 3.** Selected Cost and Clinical Outcomes from Scenario Analyses

Scenario^a^^,^^b^	IDU-IE completed treatments, %	Deaths associated with IDU-IE, %	Life expectancy, y	Discounted cost, mean, $	Incremental discounted cost, mean, $	Hospital cost, mean, $	Discounted LY	Incremental discounted LY	ICER, $ per LY^c^
No MRSA									
Usual care	77.63	5.01	73.31	416 570	NA	13 968	18.63	NA	NA
POA	82.03	4.77	73.37	412 120	4450	5360	18.66	0.0247	Cost-saving
OPAT	78.73	4.89	73.35	412 150	34	5436	18.65	Dominated^d^	Dominated^d^
Usual care/addiction care	77.58	4.86	73.35	416 990	4840	14 162	18.65	Dominated^d^	Dominated^d^
Addiction care reduces patient-directed discharge									
Usual care	77.63	5.01	73.31	416 570	NA	13 968	18.63	NA	NA
POA	86.58	4.59	73.41	414 450	1950	8610	18.68	0.0190	102 880
OPAT	82.21	4.78	73.37	412 500	4073	5516	18.66	0.0250	Cost-saving
Usual care/addiction care	87.99	4.54	73.42	417 780	3334	14 180	18.68	0.0047	716 448
Treatment uptake of POA and OPAT set at 50%									
Usual care	77.63	5.01	73.31	416 570	NA	13 968	18.63	NA	NA
POA	64.67	4.79	73.36	415 330	1240	10 960	18.66	0.0200	Cost-saving
OPAT	64.27	4.84	73.36	415 390	60	11 018	18.65	NA	Dominated^d^
Usual care/addiction care	77.58	4.86	73.35	416 990	1660	14 162	18.65	NA	Dominated^d^
Quadrupled overdose rate									
Usual care	63.33	3.21	63.37	315 000	NA	1337	14.22	NA	NA
POA	64.66	3.07	63.51	313 930	1250	972	14.29	0.0075	167 410
OPAT	63.53	3.14	63.50	312 670	2280	776	14.28	0.0593	Cost-saving
Usual care/addiction care	64.37	3.11	63.49	316 250	2320	1343	14.28	Dominated^d^	Dominated^d^
Increased uptake of addiction care and MOUD while inpatient									
Usual care	77.63	5.01	73.31	416 570	NA	13 968	18.63	NA	NA
POA	80.28	4.68	73.41	414 300	1890	8580	18.66	0.0032	581 240
OPAT	78.67	4.82	73.37	412 420	4160	5470	18.65	0.0201	Cost-saving
Usual care/addiction care	77.32	4.67	73.38	417 260	3000	14 260	18.66	0.0069	430 360
Shortened inpatient stay and increased eligibility for POA therapy									
Usual care	77.63	5.01	73.31	416 570	NA	13 968	18.63	NA	NA
POA	81.49	4.78	73.37	412 117	4454	6372	18.66	0.0240	Cost-saving
OPAT	78.73	4.89	73.35	412 150	34	5436	18.65	Dominated^d^	Dominated^d^
Usual care/addiction care	77.58	4.86	73.35	416 990	4840	14 162	18.65	Dominated^d^	Dominated^d^

Abbreviations: IDU-IE, injection drug use–associated infective endocarditis; ICER, incremental cost-effectiveness ratio; LY, life-year; MOUD, medication for opioid use disorder; MRSA, methicillin-resistant *Staphylococcus aureus*; NA, not applicable; OPAT, outpatient parenteral antimicrobial therapy; POA, partial oral antibiotic.

^a^ Scenarios assumed (1) all patients with IDU-IE were eligible to receive POA therapy, (2) addiction care services reduced the percentage of patient-directed discharges (ie, leaving the hospital against medical advice) from 5.0% to 2.5% per week, (3) the uptake of POA therapy or OPAT was limited to 50% of all patients, (4) the rate of overdose within the community and outpatient settings was quadrupled, (5) increased uptake of inpatient addiction care services and MOUD, and (6) inpatient stay was shortened to 2 weeks and eligibility to receive POA therapy was increased.

^b^ The usual care strategy comprised 4 to 6 weeks of inpatient intravenous (IV) antibiotic therapy along with opioid detoxification. The usual care/addiction care strategy comprised 4 to 6 weeks of inpatient IV antibiotic therapy along with inpatient addiction care services that offered MOUD. The OPAT strategy comprised 3 weeks of inpatient IV antibiotic therapy along with addiction care services followed by OPAT. The POA strategy comprised 3 weeks of inpatient IV antibiotic therapy along with addiction care services followed by POA therapy.

^c^ The overall incremental cost-effectiveness ratio was calculated as the difference in the mean discounted costs for the total US population divided the difference in the discounted quality-adjusted life expectancy for the total US population, all of which were discounted at 3% per year.

^d^ Cost more and had worse clinical outcomes.

Clinicians have expressed concern regarding the possibility of overdose while receiving outpatient antibiotic therapy. In a scenario quadrupling the rate of overdose in the community, our findings regarding improved outcomes with partial oral antibiotic and OPAT regimens did not qualitatively change. The OPAT strategy was the least expensive at $312 670 per person compared with the partial oral antibiotic strategy ($313 930 per person) and the usual care/addiction care strategy ($316 250 per person) and resulted in 0.059 additional LYs. The partial oral antibiotic strategy had an ICER of $167 410.

**Figure 1.**
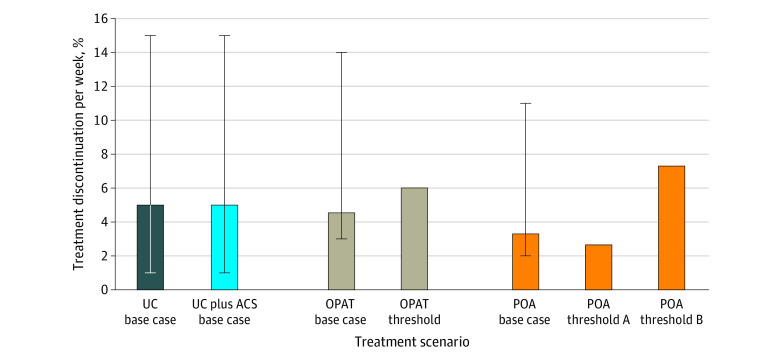
Threshold Values for Treatment Completion Results of 3 threshold analyses examining which value of treatment discontinuation per week changed the major findings. Error bars for the base case scenarios present the upper and lower ranges of the uniform distribution implemented within the probability sensitivity analyses for the partial oral antibiotic (POA) therapy and outpatient parenteral antimicrobial therapy (OPAT) strategies or the normal distribution and 1 SD range for the usual care (UC) and UC plus addiction care services (ACS) strategies. The brown bar indicating the OPAT threshold represents the threshold value (6.01% per week) for the percentage of patients discontinuing OPAT per week at which there was no longer a gain in life-years (LYs) compared with the UC base case. The orange bar indicating POA threshold A represents the threshold value (2.65%) for the percentage of patients discontinuing POA per week at which POA was cost-effective compared with OPAT at a $100 000 per LY threshold. The orange bar indicating POA threshold B represents the threshold value (7.30%) for the percentage of patients discontinuing POA per week at which there was no longer a gain in LYs compared with the UC base case.

We performed several threshold analyses. First, because uncertainty remained regarding the comparative benefit of IV vs partial oral antibiotic therapies, we performed a threshold analysis of the minimum benefit of both partial oral antibiotic and OPAT strategies, lower than which the usual care strategy provided the best outcomes (Figure 1; eFigures 1 and 2 in the Supplement). We found that the usual care strategy provided the best outcome when treatment completion was lowered from the base case of 87% to 83% for the OPAT strategy and from the base case of 90% to 80% for the partial oral antibiotic strategy. When treatment completion was lowered to 83% for the OPAT strategy and 80% for the partial oral antibiotic strategy, there was no longer a gain in LYs compared with the usual care strategy. When completion of partial oral antibiotic therapy increased to 92%, the partial oral antibiotic strategy was preferred to the OPAT strategy. Partial oral antibiotic therapy was cost-effective compared with OPAT at the $100 000 per LY threshold.

Next, we explored the rate at which patients accepted a given therapy. When OPAT uptake decreased from 100% to 79%, OPAT was no longer the preferred strategy because the mean discounted cost of OPAT ($413 860) became equivalent in cost to the partial oral antibiotic treatment strategy ($413 920). When partial oral antibiotic therapy uptake increased from 79% to 86%, partial oral antibiotic therapy was the preferred strategy, with an ICER of $72 182 per LY (eFigure 1 in the Supplement). In a threshold analysis assessing cost, when OPAT cost was $26 000 per week (compared with $1590 per week in the base case model), the OPAT strategy no longer met the $100 000 per LY willingness-to-pay threshold compared with the usual care strategy (eFigure 2 in the Supplement).

**Figure 2.**
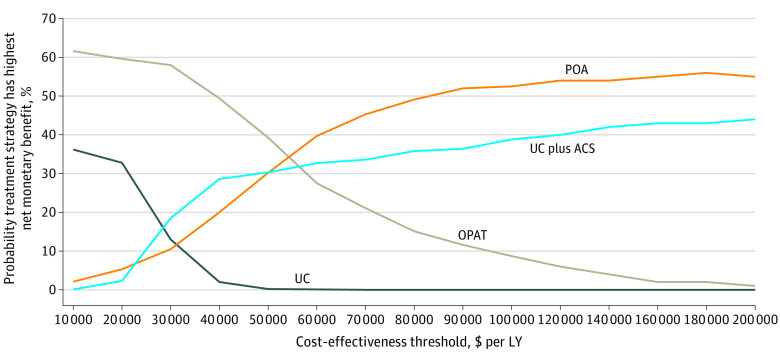
Cost-effectiveness Acceptability Curve for Injection Drug Use–Associated Infective Endocarditis Antibiotic Treatment Strategies Cost-effectiveness acceptability for the probability sensitivity analyses of the base case model. The cost-effectiveness willingness-to-pay thresholds shown on the x-axis are in 2020 US dollars. Net monetary benefit was calculated as cost subtracted from the product of the benefit multiplied by the willingness-to-pay threshold. ACS indicates addiction care services; LY, life-year; OPAT, outpatient parenteral antimicrobial therapy; POA, partial oral antibiotic therapy; and UC, usual care.

Our major findings did not qualitatively change in the probabilistic sensitivity analyses (Table 2), in which the percentages of patient-directed discharge and treatment uptake were held constant while almost all other parameters were varied (Table 1). Probabilistic sensitivity analyses were used to calculate CrI s for discounted LYs for the usual care (18.63 LYs; 95% CrI, 17.28-18.67 LYs), OPAT (18.75 LYs; 95% CrI, 17.32-18.70 LYs), partial oral antibiotic therapy (18.66 LYs; 95% CrI, 17.34-18.74 LYs), and usual care/addiction care (18.65 LYs; 95% CrI, 17.30-18.70 LYs) strategies. A cost-effectiveness acceptability curve (Figure 2) using output from the probabilistic sensitivity analyses revealed that either the partial oral antibiotic or OPAT strategy yielded the greatest net monetary benefit 100% of the time. Up to a willingness-to-pay threshold of $60 000, the OPAT strategy was preferred, and at a willingness-to-pay threshold higher than $60 000, the partial oral antibiotic strategy was preferred.

## Discussion

In this microsimulation modeling study, treatment of IDU-IE with partial oral antibiotic therapy or OPAT was associated with similar or improved long-term clinical outcomes compared with usual care while also being cost-effective. Within our base case model, we assumed that patients with IDU-IE associated with MRSA infection were not eligible to receive partial oral antibiotic therapy and, as a result, the OPAT strategy was found to be the most cost-effective. Without the exclusion of MRSA infection, the partial oral antibiotic strategy was optimal.

Up to 1 in 4 patients with IDU-IE die within 1 year after hospital admission.^70^ Challenges associated with long periods of hospitalization may be justified if hospital stays improve outcomes; however, establishment of the inferiority of alternative approaches is necessary. An increasing body of evidence suggests that OPAT and partial oral antibiotic strategies are feasible for the treatment of IDU-IE in this population, producing similar or improved clinical outcomes.^5,6^ Informed by these existing studies, we modeled the long-term outcomes associated with offering alternative antibiotic strategies paired with addiction care services and found that both the OPAT and partial oral antibiotic strategies were associated with improved outcomes compared with the usual care strategy. Our results suggest that OPAT and partial oral antibiotic regimens may be as clinically beneficial and less costly than the usual care regimen, and these findings support expanding opportunities to research and implement these options among patients with IDU-IE. Our findings also reinforce the importance of addiction care services and contribute to increasing evidence suggesting the necessity of addiction care services for the treatment of individuals with opioid use disorder.^18^

Concerns regarding the efficacy of oral antibiotic medications have hindered efforts to expand the use of partial oral antibiotic therapy for the treatment of IDU-IE. Within our main analysis, we assumed that patients with MRSA-associated IDU-IE were ineligible to receive partial oral antibiotic therapy but that otherwise the modeled antibiotic therapies had similar treatment completion rates if the treatment course was completed. Adherence to and completion of antibiotic treatment are important parameters to consider when assessing potential administration of partial oral antibiotic regimens to patients with IDU-IE. Previous studies on the implementation of care for hepatitis C viral infection among individuals who inject drugs and are receiving MOUD have reported high rates of adherence to antiviral treatment regimens that were similar to the rates of antiretroviral treatment adherence among people with HIV infection who inject drugs.^71,72^ Our threshold analysis revealed that when 80% or more of the patients receiving partial oral antibiotic therapy and 83% or more of the patients receiving OPAT successfully completed treatment, these regimens would continue to improve life expectancy compared with usual care. Although the intention of the usual care approach is universal treatment completion, the reality of noncompletion of treatment is likely underappreciated when weighing the risks and benefits of treatment strategies. Within the model, potential differences in the benefits of regimens were overcome by large differences in treatment completion. The model also assumed that a full 6 weeks of therapy was needed before treatment completion and that a mean inpatient stay of 3 weeks was needed before initiation of partial oral antibiotic or OPAT regimens. Therefore, our estimate was likely conservative.

Access to postacute care facilities for administration of OPAT may limit the ability of institutions to offer this treatment regimen.^10,22^ Postacute care facilities often refuse to accept patients with histories of active substance use despite the fact that these practices violate the Americans with Disabilities Act.^16^ However, we found within a scenario analysis that even when individuals had a very high probability of overdose after leaving the hospital, alternative antibiotic regimens were associated with improvement in outcomes compared with the usual care regimen. This finding suggests that the opportunity to complete treatment and link to MOUD through addiction care services may prevent more overdose fatalities than an extended hospital stay. These results can be used as an advocacy tool for agencies such as Medicaid to work with postacute care facilities to improve access to OPAT.

We accounted for some socioeconomic challenges, such as homelessness, by assuming that only one-half of patients could receive at-home OPAT. There are circumstances in which hospitalization may be preferable to the alternative (eg, no housing), but administration of OPAT within in a postacute care facility rather than a hospital may be preferable to both approaches. However, the high rate of adverse events associated with OPAT, including peripherally inserted central catheter line infection and thrombosis, will need to be considered when discussing alternative antibiotic therapy strategies.^9^ Clinicians may consider engaging in patient-centered decision-making when offering these treatment strategies, with housing not used as the sole determining factor when selecting an antibiotic treatment strategy.

### Limitations

This study has several limitations. First, we relied on a single published study to inform parameters on partial oral antibiotic treatment completion and regimen costs. However, a prospective cohort study^73^ examining the efficacy of a partial oral antibiotic regimen for patients with early patient-directed discharge is currently being conducted, and model parameters are within the currently observed range. Second, although important model parameters were informed by studies of the target population, unmeasured confounders may have impacted the results of these studies. Despite these limitations, our findings did not qualitatively change in sensitivity analyses and when varying assumptions were used, which may enable clinicians and hospital staff to consider these findings within their local context.

## Conclusions

Results from this decision analytical modeling study suggest that, if implemented, the strategies could save the health care system a substantial amount of money in lifetime hospitalization costs alone for the estimated 750 000 individuals currently injecting drugs in the US.^26^ Those savings could be shifted to programs that specifically address the opioid epidemic, such as initiatives to improve access to MOUD, promote safer injection techniques, and provide multidisciplinary outpatient support systems, including peer navigators and case managers, to decrease the future incidence of IDU-IE and support patient retention in substance use disorder care programs.

## Notes

Supplement. **eMethods 1.** Introduction, REDUCE Model, Cohort Initiation, and Sequelae of Drug Use **eMethods 2.** Inpatient Hospitalization, Outpatient Care, Behavioral Transitions, and Mortality **eMethods 3.** Outpatient Parenteral Antimicrobial Therapy and Partial Oral Antibiotics Regimen **eMethods 4.** Model Scenarios, Scenario Analyses, Threshold Analyses, and Probabilistic Sensitivity Analyses **eTable 1.** Initializing Cohort Characteristics **eTable 2.** Calibration Targets for Incidence of Endocarditis and Fatal Overdose **eTable 3.** Weekly Probability of Developing Infective Endocarditis, Stratified by Injection Behavior Profile **eTable 4.** Hospital-Based Services **eTable 5.** Outpatient Addiction Services **eTable 6.** Model Parameters Implemented With Outpatient Parenteral Antimicrobial Therapy Module **eTable 7.** Estimated Weekly Cost of Medication for Outpatient Intravenous Antibiotic Therapy **eTable 8.** Estimated Weekly Cost of Treatment Services Related to Outpatient Parenteral Antimicrobial Therapy **eTable 9.** Model Parameters Implemented With Partial Oral Antibiotic Therapy Module **eTable 10.** Estimated Weekly Cost of Medication for Outpatient Oral Antibiotic Therapy **eTable 11.** Estimated Weekly Cost of Treatment Services Related to Partial Oral Antibiotic Therapy **eTable 12.** Key Input Parameters for Treatment Strategies Modeled Within REDUCE to Compare Approaches to Treating Drug Use–Associated Endocarditis **eTable 13.** Cost-effectiveness Outcomes From Threshold Analyses **eFigure 1.** Threshold Values for Treatment Uptake of Partial Oral Antibiotic Therapy and Outpatient Parenteral Antimicrobial Therapy **eFigure 2.** Threshold Values for Weekly Cost of Treatment for Partial Oral Antibiotic Therapy and Outpatient Parenteral Antimicrobial Therapy
eReferences
