# Supplementary material for: Simulated Cost-effectiveness and Long-term Clinical Outcomes of Addiction Care and Antibiotic Therapy Strategies for Patients With Injection Drug Use–Associated Infective Endocarditis
Source: JAMA Netw Open. 2022 Feb 28;5(2):e220541. doi: 10.1001/jamanetworkopen.2022.0541 (PMC8886538; doi:10.1001/jamanetworkopen.2022.0541)
Supplement: Supplement. — eMethods 1. Introduction, REDUCE Model, Cohort Initiation, and Sequelae of Drug Use eMethods 2. Inpatient Hospitalization, Outpatient Care, Behavioral Transitions, and Mortality eMethods 3. Outpatient Parenteral Antimicrobial Therapy and Partial Oral Antibiotics Regimen eMethods 4. Model Scenarios, Scenario Analyses, Threshold Analyses, and Probabilistic Sensitivity Analyses eTable 1. Initializing Cohort Characteristics eTable 2. Calibration Targets for Incidence of Endocarditis and Fatal Overdose eTable 3. Weekly Probability of Developing Infective Endocarditis, Stratified by Injection Behavior Profile eTable 4. Hospital-Based Services eTable 5. Outpatient Addiction Services eTable 6. Model Parameters Implemented With Outpatient Parenteral Antimicrobial Therapy Module eTable 7. Estimated Weekly Cost of Medication for Outpatient Intravenous Antibiotic Therapy eTable 8. Estimated Weekly Cost of Treatment Services Related to Outpatient Parenteral Antimicrobial Therapy eTable 9. Model Parameters Implemented With Partial Oral Antibiotic Therapy Module eTable 10. Estimated Weekly Cost of Medication for Outpatient Oral Antibiotic Therapy eTable 11. Estimated Weekly Cost of Treatment Services Related to Partial Oral Antibiotic Therapy eTable 12. Key Input Parameters for Treatment Strategies Modeled Within REDUCE to Compare Approaches to Treating Drug Use–Associated Endocarditis eTable 13. Cost-effectiveness Outcomes From Threshold Analyses eFigure 1. Threshold Values for Treatment Uptake of Partial Oral Antibiotic Therapy and Outpatient Parenteral Antimicrobial Therapy eFigure 2. Threshold Values for Weekly Cost of Treatment for Partial Oral Antibiotic Therapy and Outpatient Parenteral Antimicrobial Therapy eReferences [file jamanetwopen-e220541-s001.pdf]

Supplementary Online Content

Adams JW, Savinkina A, Hudspeth JC, et al. Simulated cost-effectiveness and long-term clinical outcomes of addiction care and antibiotic therapy strategies for patients with injection drug use–associated infective endocarditis. *JAMA Netw Open*. 2022;5(2):e220541. doi:10.1001/jamanetworkopen.2022.0541

eMethods 1. Introduction, REDUCE Model, Cohort Initiation, and Sequelae of Drug Use

eMethods 2. Inpatient Hospitalization, Outpatient Care, Behavioral Transitions, and Mortality

eMethods 3. Outpatient Parenteral Antimicrobial Therapy and Partial Oral Antibiotics Regimen

eMethods 4. Model Scenarios, Scenario Analyses, Threshold Analyses, and Probabilistic Sensitivity Analyses

eTable 1. Initializing Cohort Characteristics

eTable 2. Calibration Targets for Incidence of Endocarditis and Fatal Overdose

eTable 3. Weekly Probability of Developing Infective Endocarditis, Stratified by Injection Behavior Profile

eTable 4. Hospital-Based Services

eTable 5. Outpatient Addiction Services

eTable 6. Model Parameters Implemented With Outpatient Parenteral Antimicrobial Therapy Module

eTable 7. Estimated Weekly Cost of Medication for Outpatient Intravenous Antibiotic Therapy

eTable 8. Estimated Weekly Cost of Treatment Services Related to Outpatient Parenteral Antimicrobial Therapy

eTable 9. Model Parameters Implemented With Partial Oral Antibiotic Therapy Module

eTable 10. Estimated Weekly Cost of Medication for Outpatient Oral Antibiotic Therapy

eTable 11. Estimated Weekly Cost of Treatment Services Related to Partial Oral Antibiotic Therapy

eTable 12. Key Input Parameters for Treatment Strategies Modeled Within REDUCE to Compare Approaches to Treating Drug Use–Associated Endocarditis

eTable 13. Cost-effectiveness Outcomes From Threshold Analyses

eFigure 1. Threshold Values for Treatment Uptake of Partial Oral Antibiotic Therapy and Outpatient Parenteral Antimicrobial Therapy

eFigure 2. Threshold Values for Weekly Cost of Treatment for Partial Oral Antibiotic Therapy and Outpatient Parenteral Antimicrobial Therapy

eReferences

This supplementary material has been provided by the authors to give readers additional information about their work.

© 2022 Adams JW et al. *JAMA Network Open*.

eMethods 1. Introduction, REDUCE Model, Cohort Initiation, and Sequelae of Drug Use

Introduction

The analyses reported in the main manuscript use the Reducing Infections Related to Drug Use Cost Effectiveness (REDUCE) Model of acquisition and treatment for bacterial infections and overdose associated with injection drug use. The REDUCE model tracks several clinical outcomes including number of people with infective endocarditis (IE) and overdose (OD) (otherwise known as 'sequelae'), number of cases identified, number linked to inpatient and outpatient care, number of people initiating therapy, and number achieving cure from their sequelae of drug use. The model also tracks sequelae-related mortality, quality of life, undiscounted life expectancy, discounted quality-adjusted life expectancy (QALE), discounted lifetime medical costs from the health system perspective, and non-discounted program costs from the payer perspective (for interventions designed to improve follow-up). This technical appendix provides details on key features of the model and modeling approach used for this analysis. We constructed the model and performed analyses using C++ and R (3.2.2). The model has been previously described in the peer-reviewed literature.[1] The model is available for review upon discussion with the authors and as resources are available. We did not use every component of the model for the current analysis. In addition, we provide figures and several tables detailing input parameter values and additional results cited in the manuscript.

REDUCE model

The REDUCE model is an individual-based, stochastic simulation model of the natural history of injection drug use designed to estimate the outcomes and costs associated with various strategies of prevention, treatment, and improving drug use-related care. The model uses a cycle length of one week.

Overview

The model is designed as a number of modules through which simulated individuals pass. Briefly, a cohort module helps to "create" the population of interest. Next, individuals created during cohort generation enter the "sequelae of drug use (SDU)" module, which is where they encounter probabilities of fatal or nonfatal overdose, infective endocarditis, or skin and soft tissue infections. From the SDU module, individuals enter back into the simulation or link to the "inpatient" module. In the "inpatient" module, individuals are hospitalized for their SDU. There are a variety of interventions (beyond standard hospital treatment) that individuals may encounter if those services are turned "on" by the user. Following the inpatient module, individuals have a probability of linking to outpatient care in the "outpatient" module. Linkage to outpatient care may vary based on the type of services an individual encountered in the hospital and/or the type of SDU they have (overdose vs infection). They may unlink from the outpatient module or never enter it (based on probabilities). The "behavioral transitions" module is when individuals have the probability of moving between injection frequency drug use states (high frequency, low frequency, or no current drug use), between sterile injection practice states (skin cleaning or no skin cleaning), and sharing/reusing needles. After the "behavioral transitions" module, individuals move to the "mortality, cost, and quality of life" module. At this point, the model begins again in cycle n+1.

Cohort initiation

When the model is initiated, a cohort of individuals is generated using 6 parameters:

(1) ever injection drug use status (ever/never)
(2) age (0-99)
(3) sex (M/F)
(4) injection frequency (high/low/no current/never)
(5) reusing/sharing equipment (yes/no/never).
(6) sterile injection practice (cleaning/no cleaning/never)

© 2022 Adams JW et al. *JAMA Network Open*.

From these parameters, the initializing cohort includes people who have “ever” or “never” injected drugs. Those who are “ever” injectors are stratified by injection frequency and injection practices. The model is structured such that first the user specifies the proportion of the population that has ever injected drugs. Following that, there are two methods by which the model can draw age and sex. The first is by using age/sex tables and the second is by directly specifying age and sex distribution parameters. In the latter method of drawing from age and sex, the user inputs values directly into the deterministic parameter file. These inputs include proportion male, average male age, standard deviation male age, average female age, standard deviation female age, and minimum age.

Next, among those who are ever drug users, the probability of injection frequency is drawn from an age/sex stratified table—high, low, and no current injection drug use. Within the literature, injection frequency is usually reported as summary of behavior within the past month and while frequency may change daily depending on drug availability, we assume that overall frequency is stable over a one-week period. For generating the frequency of injection, all three probabilities for an age/sex group equals 1 and the model draws from this set of probabilities. Finally, all persons who are “ever” drug users, are assigned an initial status of being a skin cleaner and a needle sharer which does not depend on age and gender. While these are the initial attributes, all individuals have the possibility of changing attributes as they move through the model. All never drug users are assigned “never” injection frequency, skin cleaning and needle sharing status.

Assumptions built into the model for the initial cohort:

1) no one starts on treatment for opioid use disorder
2) no one starts out with a history of overdose
3) no one starts with a history of infection
4) no one begins in care or in the hospital setting.

For the present analysis, we initialized a cohort representing a national sample of people with injection drug use with the characteristics presented in e**Table 1**. Population characteristics were based on the U.S. Census and the published literature.

Sequelae of drug use

Once the cohort is initialized and each individual has been assigned an initial drug use status, age, sex, and injection frequency and practices, individuals enter the sequelae of drug use (SDU) module. Broadly, the SDU in this model include infective endocarditis (IE) and overdose (OD). When they first enter, the model checks their ever/never status. If they are “never,” then they return to the simulation. Therefore, only “ever” drug users can progress through this module. The model then checks their injection frequency. If they are “no current,” then they return to the simulation. Therefore, only “low frequency” and “high frequency” injectors progress through this module. Additionally, if the individual is currently in inpatient care, they return to the simulation. If a person is currently on antibiotics, they progress through the SDU module but they cannot acquire a new infection (IE).
At this point, remaining individuals are subject to probabilities for acquiring an SDU. On the first cycle of this model, no one has a history of SDU, but have the possibility of acquiring one or multiple through their life. History of SDU is tracked as it has implications for future SDU. One assumption of the model is that SDUs can only be acquired while not “inpatient” or on antibiotics (next module).

Individuals who are eligible for an SDU, progress through a number of probabilities of acquiring an SDU. All SDUs are stratified by injection frequency (high and low) and the infectious SDU are also stratified by injection practices (skin cleaning, needle sharing). SDU probabilities are not stratified by age and sex. The model is structured such that an individual first encounters a combined probability of overdose (fatal + nonfatal), stratified by injection frequency. If an individual has a current infection their overdose rate is multiplied by the current infection multiplier. A proportion of overdoses are fatal and a proportion are nonfatal. One aspect of the model is that at this point, if a person draws a fatal overdose then they are flagged as “dead, fatal overdose.” They continue to proceed through the rest of the modules but cannot

© 2022 Adams JW et al. *JAMA Network Open*.

acquire any further attributes (e.g., they cannot get another infection, be hospitalized, start MOUDs, change their behaviors). These individuals, however, accrue the full costs of the cycle (based on background costs, costs of fatal overdose, and costs of any other SDUs that are untreated) and utilities (based on age, sex, and other current health states at the end of the cycle). For those that have a nonfatal overdose or do not have an overdose, they then face a combined probability of IE, stratified by injection frequency, skin cleaning and needle sharing attributes. The model is structured to account for a history of SDU (treated in hospital, resolved because it was a nonfatal OD) and for existing SDUs. An existing SDU is anything that an individual has during the current cycle. From a clinical perspective, this represents an "untreated" infection (e.g., someone has not gone to the hospital for their endocarditis or someone is currently on outpatient antibiotics but not cured) or a current nonfatal overdose. Once treatment is complete or the SDU resolves (as is the case with nonfatal overdose which resolves in 1 cycle), then the person is flagged with a history of the corresponding SDU.

An existing SDU causes a change in the likelihood of another SDU. In the model, there is a single multiplier for one or more existing SDUs that is applied to both the probability of OD and the probability of infectious SDU. This multiplier exists until the individual is treated for the SDU. For those who have a nonfatal overdose, the existing SDU multiplier will be applied to the probability of infection in the same cycle only since an existing nonfatal OD (that does not link to inpatient), only lasts one cycle. Additionally, a history of SDUs changes the probability of future SDUs. Multipliers are only applied to the SDU for which there is a history (e.g., OD history changes the probability of recurrent OD; any infection history changes the probability of future infection [any infection, not just the one that occurred]). For OD, there are 4 multipliers (e.g., 1 past nonfatal OD, 2-3 past nonfatal OD, 4-7 past nonfatal OD, and 8+ past nonfatal ODs). For history of treated infections, there is only one multiplier (1+ past treated infections). For instance, in cycle 1, an individual gets IE but does not go to the hospital/receive treatment and does not die in cycle 1. By cycle 2, having IE makes that individual have a greater probability of OD. For this model, individuals will not be able to acquire the same SDU in that next cycle. From the previous example, the individual with IE will only be able to acquire OD, not IE in cycle 2. While that infection remains untreated, there is an effect on getting another infection/OD. Once that infection is treated, then there is a separate effect of this infection on future infections. Therefore, this module has two multipliers: 1) one that can change the probability of an additional SDU if current SDU is untreated, and 2) one that can change the probability of a recurrent SDU (in the future) if the current SDU is fully treated and they survive it.

If an individual does not acquire an SDU in the current cycle and does not have an untreated SDU from a past cycle, they return to the simulation. If they acquire one or more SDUs, or have an untreated SDU from a past cycle, then individuals draw linkage probability to inpatient from the SDU. Linkage to inpatient depends on the linkage probability of their SDU; if an individual has more than one SDU, their linkage probability is the highest of the linkage probabilities for the SDUs they have. There remains the possibility that an individual does not link to inpatient. In the case of nonfatal OD, it implies that the OD was not severe enough to require hospitalization (or was treated in the field). In the subsequent cycle, there should not be a flag for untreated overdose. All nonfatal overdoses are, by definition, treated so the "existing" state can only last for the cycle in which the non-fatal overdose occurs. In the case of endocarditis, the untreated flag should remain on until the person either dies or links to inpatient care and gets cured. This is because endocarditis is generally uniformly fatal if untreated. Individuals who go to the hospital will be classified as "inpatient" starting in the same cycle and will have an "in-hospital mortality." Once they leave the hospital, they are considered as having a history of infection. If an individual does not link to inpatient, they are classified as having "existing" SDU and have different risks of death (untreated mortality probabilities for each SDU). Individuals who come to the SDU module on subsequent cycles with an additional SDU (>1 SDU at a time) will have the probability of hospitalization that is equal to the highest probability of the SDUs.

Attributes that an individual can acquire in this module and are tracked:

© 2022 Adams JW et al. *JAMA Network Open*.

1. Current IE
2. Current OD, non-fatal
3. Current OD, fatal
4. History of treated IE
5. History of treated OD

© 2022 Adams JW et al. *JAMA Network Open*.

eMethods 2. Inpatient Hospitalization, Outpatient Care, Behavioral Transitions, and Mortality

Inpatient hospitalization

One assumption of the model is that any individual that has either a) current injection drug use or b) a current, untreated SDU is presumed to have opioid use disorder (OUD). Some sequelae of OUD are infectious and some are non-infectious (e.g., overdose).

Each individual with 1+ SDU has a probability per cycle of presenting to an inpatient setting for their care. When individuals enter the inpatient module, the model checks their current SDU status. If they do not have a current untreated SDU or died of fatal overdose in the previous module, or they are on outpatient antibiotics, then they return to the simulation. Therefore, only those individuals with active SDU can progress through this module.

The path through the inpatient module is conditional on the SDU(s) that an individual has: nonfatal OD, IE, or combination. The hospitalization duration for overdose is 1 cycle; the hospitalization duration for IE is drawn stochastically from a normal distribution with a user defined mean and standard deviation; the model allows for a maximum hospitalization to be set so that at the end of the max amount of time a person will leave the hospital. Each hospitalization is associated with a cost that is accrued in later module. The key feature of this module is that individuals may encounter a variety of in hospital services. These services are either turned on or off by the user depending on the analysis. If they are on, then individuals will have a probability of being offered and of accepting those services during their hospitalization. Each service has an effect either within this module or elsewhere in the simulation. Each service is associated with a cost that is applied in a separate module at the end of the simulation. These interventions are applied only in the last cycle of hospitalization and they will have post-treatment effective cycles drawn from a normal distribution. Individuals should be “marked” as using/receiving a service such that the cost can be tabulated in the separate module. Additionally, some of the services have an independent effect on quality of life. Similar to cost, this is applied in a separate module at the end of the simulation. Hospitalization is associated with a decreased QoL so there is a hospitalization QoL weight that can be applied in a separate module at the end of the simulation.

Each individual has a probability of in-hospital mortality that is discussed in detail in the mortality section. It is mentioned here to note that it is an attribute that an individual can acquire. During hospitalization, individuals “carry” a flag/marker that designates them as hospitalized. While hospitalized, individuals cannot get a new SDU so they will not enter SDU module. They have an “in hospital” mortality that is conditional on the SDU for which they are hospitalized. For the duration of their hospitalization, their injection frequency is considered to be “no current” regardless of their actual status and they are not exposed to behavior transitions. The exception to this rule is as follows: in the last hospitalization cycle, individuals are exposed to behavior transitions based on their pre-hospitalization status. If they have received any intervention that would affect their behaviors (MOUD, skin cleaning education or clean needle distribution), the intervention effect will be applied to their actual or pre-hospitalization behaviors and post-treatment effective cycles will be drawn. These behavioral changes are assigned in the last inpatient cycle so that they take effect the first cycle out of inpatient. However, cost-life-mortality module still consider them as “no current”. We assume that 5% of patients leave against medical advice (AMA) per week or as a patient-directed discharge prior to completion of treatment, informed by published studies reporting a high rate of AMA within this patient population.[10, 11] When the inpatient hospitalization time has lapsed or patients leave against medical advice, individuals move to the outpatient module. In the outpatient module, they have a probability of then linking to different types of care.

Outpatient care

There are two different ways by which an individual can enter the outpatient module. First, an individual can enter via background linkage. This means that those who are not hospitalized but "decide" to seek care can do so by entering this module. Second, an individual can enter via the inpatient module.

For individuals entering from the simulation (background). Each individual encounters the outpatient module. Individuals with a "death" flag from a previous module (fatal overdose) enter the outpatient module and immediately return to the simulation. Individuals who are currently hospitalized immediately return to the simulation. All other "ever" drug user individuals have a probability of linking to outpatient care and progress through the outpatient module, regardless of history of SDU or drug use status. If individuals do not draw "linkage" then they return to the simulation.

For individuals entering from the inpatient module (inpatient linkage). When the inpatient hospitalization time has lapsed, then individuals encounter a linkage probability to the outpatient module depending on inpatient services they have received.

Outpatient addiction care. Individuals have a probability of linking to outpatient addiction care (either with or without MOUDs). One cannot be simultaneously in outpatient addiction care with MOUDs and without MOUDs (these are separate states). But individuals can be simultaneously in outpatient addiction care (with or without MOUDs) and outpatient antibiotics.

Individuals have a probability of unlinking from outpatient addiction care either with or without MOUDs or transitioning between MOUD states. There is a separate probability of linking to outpatient addiction care (with or without MOUDs) for those coming from the inpatient module and those coming from the simulation (spontaneous linkage/background linkage). There are different linkage probabilities for the following groups:

1. Individuals who have received inpatient addiction care but did not get MOUD
2. Individuals who have received inpatient addiction care and got MOUD
3. Individuals who did not receive inpatient addiction care but got inpatient MOUD
4. Individuals who did not receive any relevant inpatient services or individuals coming from the background (no hospitalization)

If an individual is in outpatient addiction care and acquires an infection they will automatically be linked to inpatient care in the next cycle. In this case, they will unlink from outpatient care and all outpatient related flags/cycles will be cleared. For the present analysis, we added two outpatient antibiotic strategies which are described in detail below under the sections titled "Outpatient parenteral therapy" and "Partial oral antibiotic therapy".

Behavioral transitions

Following the inpatient and outpatient modules, individuals move to the behavioral transitions module. Individuals may also enter this module "from the simulation." The latter represents the ability of someone to change their behaviors organically (without interventions). This is the module in which they can move between high frequency, low frequency, and no current use states, move from never and ever IDU, move between skin cleaning and not skin cleaning states, and move between sharing needles and not sharing needles states. There is a prior probability of movement between states (status quo) and various "flags" acquired throughout the model progression that impact certain probabilities. These have been outlined in various other module descriptions but are also be outlined below.

Treatment Effects: The primary driver of morbidity and mortality in the module is the injection frequency. High frequency individuals are at higher risk than low frequency injectors of sequelae of drug use (SDUs), which include overdose, skin/soft tissue infections, and endocarditis in this model. All persons who are

© 2022 Adams JW et al. *JAMA Network Open*.

“ever” injectors have the possibility of moving to a higher or lower injection frequency state (depending on their current state) or staying in their current state per cycle. For instance, a high frequency injector may remain as a high frequency injector or may move to low frequency or no current use states. There are a few ways that the injection frequency can be modified in the model.

Mechanisms by which transitions between injection frequency states are changed:

1) Hospitalization.
2) Outpatient MOUD initiation.
3) Inpatient MOUD initiation.
4) Behavioral transitions with MOUD.

Mortality

There are two places in the model that an individual can die: fatal overdoses in the SDU module and in the mortality module. To review, in the SDU module, an individual draws a combined probability of all types overdose which is stratified by injection frequency (high and low frequency). From that combined probability, an individual can draw either a fatal or non-fatal overdose. If an individual draws a fatal overdose, they go through the remainder of the cycle with a “fatal OD” flag up which does not allow them to get any further interventions, collect additional costs, change their behavior status, etc., however, they will accumulate the background cost and utility of that cycle. As such, the background mortality in the mortality module should exclude overdose mortality.

The background mortality risk is an age and sex adjusted mortality probability (excluding fatal overdose). There are a number of occurrences in the model that can impact the weekly risk of mortality. First, individuals who are hospitalized for an SDU (non-fatal overdose or endocarditis) have an increased risk of death. If the inpatient individual receives an ID consult, their infection inpatient mortality rate is augmented by an ID consult mortality multiplier (ID consult will not affect overdose mortality). Second, individuals who have an untreated skin and soft tissue infection or untreated infective endocarditis have an increased risk of death. These risks are input as probabilities (and converted to rates by the model) which are then *added* to the background mortality at the end of each cycle. Once a patient is cured of their infection, their SDU flags are removed and their mortality goes back to background mortality. The mortality risk only applies for each cycle that they have that risk. For example, a person gets endocarditis and does not present to inpatient care during a cycle. Then they have an “existing endocarditis” flag that the end of the cycle should prompt the rate of death for untreated endocarditis to be added to the background mortality. On cycles 2-5 that same individual, however, is hospitalized and being treated for their endocarditis. For those cycles, they get an “in-hospital for endocarditis” flag such that the in-hospital endocarditis mortality rate is added to their background mortality each cycle. On cycle 6, this person leaves the inpatient setting (completes treatment) so all flags are, therefore, off and at the end of that cycle they get only background mortality. We do not include an additional mortality risk for being an active drug user since most of that risk will be folded into overdose and other SDUs.

Cause of death as an output: In the model, individuals can die of background causes or as a direct result of their injection drug use. Direct causes of injection drug use include:

1. Overdose (combination of fatal overdose/ hospitalized and nonfatal OD that dies in the hospital)
2. Endocarditis (combination of hospitalized and non-hospitalized)

Aside from fatal overdose, all of the other causes of death get added to the background mortality as outlined above. For instance, an individual’s weekly probability of death (conditional on not dying of a fatal overdose) may be p_d and they may have endocarditis which increases their risk of death by *x*. The individual’s weekly risk of death is, therefore, *the sum of the rates converted to a probability.* However, as an output, we need to be able to determine the attributable cause of death (this person may have died of

endocarditis OR background causes). To do this, we use the sum of the rates as the denominator and the individual mortality risk (rates) as the numerator in drawing the cause of death. Important for consistency, the input parameters are probabilities and therefore all rates are calculated in the model.

Costs
Costs are accrued for a variety of reasons. At the end of each cycle, costs associated with certain characteristics are added to the background costs. All costs and life expectancy have a discount rate applied at the end of the cycle so that we can derive a discounted cost and a discounted life expectancy.

1. Background costs: age and sex stratified costs of being alive which are the same for never and ever IDUs
2. Injection drug use costs: Ever injection drug users should have costs that are stratified by frequency:
    a. Cost of no current injection drug use
    b. Cost of high frequency injection drug use
    c. Cost of low frequency injection drug use
3. Cost of fatal overdose
4. Cost of non-fatal overdose not hospitalized
5. Cost of untreated endocarditis
6. Cost of untreated skin/soft tissue infection
7. Per cycle costs of hospitalization for endocarditis*
8. Per cycle costs of hospitalization for skin and soft tissue infection*
9. Per cycle costs of hospitalization for overdose*
10. Inpatient services costs
    a. Addiction consult service: recurring weekly cost while inpatient
    b. MOUDs: recurring weekly cost while inpatient
11. Outpatient services costs
    a. Outpatient addiction with MOUD: recurring weekly cost while on MOUD and linked to addiction care
    b. Outpatient addiction without MOUD: recurring weekly cost while linked to care

*If someone is hospitalized for multiple causes (IE, OD) they do not get costs for both as we would be double counting. Instead, they get the maximum of the hospitalization costs for what they have (i.e., if the individual was hospitalized for OD and IE, they would receive whichever costs are higher, OD or IE, but not costs for both).

© 2022 Adams JW et al. *JAMA Network Open*.

eMethods 3. Outpatient Parenteral Antimicrobial Therapy and Partial Oral Antibiotics Regimen,

Outpatient parenteral antimicrobial therapy (OPAT)

Outpatient parenteral antimicrobial therapy (OPAT) is widely used to treat infections requiring prolonged antibiotic therapy with a proven safety record.[12] Several recent studies have provided an evidence regarding safety of OPAT for persons with injection drug use (PWID).[13-15] Suzuki et al. performed a systematic literature review to evaluate the safety and effectiveness of OPAT among PWID.[13] Six studies were U.S.-based. In general, patients were discharged to home following hospital admission; however, studies also reported discharge to a medical respite facility, skilled nursing facility, residential treatment facility, and a group home. Outcomes on treatment completion, mortality, and active substance use following admission were used to parameterize the OPAT treatment module.

Percent uptake

For the main analysis, we assumed that all patients admitted with DUA-IE would be eligible for OPAT at a one point in their treatment. If the probability of discharge on OPAT by 6 weeks is 99%, the weekly probability of discharge on OPAT is 53.6% (**eTable 6**). We lowered this percent to 50% within a scenario analysis described within the "Scenario analyses" subsection.

Duration of treatment

Fannuchi et al. conducted a pilot randomized trial comparing usual care (IV antibiotics in the hospital) to receiving combined OPAT and MOUD for persons with OUD hospitalized with a IDU-associated infection.[15] The reported average length of hospital stay was 22.4 (SD=7.1) for OPAT participants compared to 45.9 (SD=7.8) for usual care participants. All 10 participants assigned OPAT completed treatment which involved an average of 20.1 (SD=11.1) days of outpatient antibiotics. On average, OPAT involved a 3 week stay within the hospital followed by 3 weeks outpatient antibiotics within the model.

Treatment discontinuation and readmission

Every week following hospitalization and prior to the completion of the antibiotic regimen (with a mean duration of six weeks), there is a weekly probability of unlinking from antibiotics. This represents both treatment failure as well as a patient voluntarily discontinuing. If the antibiotic regimen is not fully completed (i.e., patient unlinks from antibiotics at four weeks rather than six), there is a 100% probability of relapse of infection and the patient will be re-admitted or die.

To parameterize the probability of unlinking from OPAT following an inpatient stay for DUA-IE, we used data from Fanucchi et al. and a recent study by D'Couto et al.[14] We calculated the mean percentage of treatment completion reported by D'Couto et al. for participants who were discharged to home on OPAT (81% completed treatment) as well as Fanucchi et al. (100% completed treatment) and weighted by sample size to calculate that 87% of patients initiating OPAT with an offer of ACS and MOUDs complete treatment. We used the inverse and re-scaled to calculate a weekly probability of discontinuing treatment and readmission (4.54% per week).

For a range around this estimate, we used the systematic review from Suzuki et al. which found that OPAT completion rates of predetermined duration ranged from 64% to 91% in U.S. based studies.[13] We converted these to an inverse probability to represent the probability of discontinuing treatment and then to weekly probabilities (3-14%).

© 2022 Adams JW et al. *JAMA Network Open*.

Cost

Costs included within the model include the cost of medication (antibiotics) as well as treatment utilization, including physician visits and typical laboratory testing.

Pharmaceutical costs

To specify antibiotic regimens and estimate associated costs, we first estimated the distribution of organisms leading to DUA-IE. Rodger et al. reported on 202 first-episode cases of DUA-IE and found that *staphylococcus aureus* infections were the causative organism in 77.2% of cases in PWID (156 of 202), followed by 6.4% (13 of 202) with a polymicrobial infection, and 5.4%(11 of 202) caused by enterococci.[16] Hartnett et al. reported that 11% of drug-use associated infections (not just but inclusive of DUA-IE) were caused by streptococci.[17] Infectious disease physicians on our study team examined the data from both studies and estimated that DUA-IE infections were due to the following organisms: 56% Methicillin-sensitive *Staphylococcus aureus*, 21% Methicillin-resistant *Staphylococcus aureus*, 11% streptococci, 6% enterococci, and 6% other (polymicrobial, culture negative, pseudomonas or acinetobacter, etc.).

Using this distribution of organisms, we then used recommended regimens based on guidelines published by the American Heart Association[18] to assign the most likely IV antibiotic regimen (see **eTable 7**). We then looked up medication costs using the Federal Supply Schedule (https://www.va.gov/opal/nac/fss/pharmPrices.asp, accessed 2/23/2021). If multiple prices were reported for the same medication, we averaged the price and used the costs to create a range for the probabilistic sensitivity analyses. For the estimated cost for OPAT, we calculated a weighted average daily cost using the following formula:

0.21($Vancomycin)+0.56($Cefazolin)+0.11($Penicillin)+0.06($Ampicillin NA)+0.06($Ceftriaxone)

We then multiplied this by 7 to calculate the average weekly cost resulting in an estimated weekly cost of medication of $133.00 ($124.95-143.36).

Treatment costs

Before discharge, patients who go on to receive OPAT have a PICC line placed as well as a chest x-ray which leads to an inpatient cost of $126.46.

Following discharge from inpatient care, patients with DUA-IE could receive OPAT at home or in a skilled nursing facility, rehabilitation center, or another post-acute care facility. Costs of treatment per day within a post-acute care facility were estimated using data from Boston Medical Center (see **eTable 8**) and includes nursing visits, laboratory testing, and physician visits.

Treatment costs associated with receiving OPAT at home were derived from studies reporting typical treatment services received during OPAT at home.[19, 20] We assumed that, on average, home infusion would lead to the following treatment costs: physician visit every two weeks, weekly nurse visit, weekly complete blood count (CBC) with differential, weekly liver function testing, and weekly blood urea nitrogen and creatinine testing. In addition, we assumed that 10% of patients on OPAT would require a CT angiogram and 5% of patients would require an echocardiogram due to suspected septic emboli or other complications based on expert opinion. Costs were calculated in 2020 USD using the Physician Fee Schedule and Laboratory Fee Schedule (both accessed on 4/19/2021). On average, the cost of home infusion per week was $469.10 ($461.05-479.46), including costs of antibiotics as calculated above. This is in line with the cost estimate included within a report to Congress from MedPac on Medicare coverage and payment for home infusion therapy which stated an average gross drug cost per user of $1,250 per person (includes bundled payment of drug and equipment but no nursing visits) or $417 per week if assuming OPAT for three weeks.

© 2022 Adams JW et al. *JAMA Network Open*.

To estimate an overall weighted weekly cost of OPAT, we assumed that 50% of patients discharged on OPAT would have home infusion therapy and 50% would receive OPAT at a post-acute facility. Data received from Boston Medical Center indicated that 50% of patients with DUA-IE were homeless, and therefore, could not be discharged home. We also assumed that percentage of patients would prefer or require the additional support of a post-acute facility. We used cost data from patients with DUA-IE staying at post-acute facilities to parameterize the average weekly cost of OPAT at a post-acute facility. The average weekly cost at a post-acute facility was $2,569.00 with a minimum weekly cost of $637.00 and maximum weekly cost of $11,613. We then added the cost of antibiotics to calculate the total cost. We assumed that 50% of OPAT recipients were discharged home and 50% went to a post-acute facility for care.

Partial oral (PO) antibiotic regimen

Percent uptake

For the main analysis, we assumed that only patients admitted with DUA-IE with non-MRSA organisms would be eligible for partial oral antibiotics. In a study of 202 first-episode DUA-IE, Rodger et al. reported that methicillin-resistant *staphylococcus aureus* infections were the causative organism in 21.3% of cases.[16] Therefore, we assumed that 79% patients admitted with DUA-IE would be eligible for PO at a one point in their treatment. If the probability of discharge on PO by 6 weeks is 79%, the weekly probability of discharge on PO is 22.9%. To explore the implications of a lower percentage of MRSA organisms. For these analyses, we ran a scenario analysis where we assumed that all DUA-IE cases were non-MRSA.

Duration and rate of treatment completion

Every week following hospitalization and prior to the completion of the antibiotic regimen (with a mean duration of six weeks), there is a weekly probability of unlinking from antibiotics. This represents both treatment failure as well as a patient voluntarily discontinuing. If the antibiotic regimen is not fully completed (i.e., patient unlinks from antibiotics at four weeks rather than six), there is a 100% probability of relapse of infection and the patient will be re-admitted or die.

To parameterize the probability of unlinking from partial oral therapy following an inpatient stay for DUA-IE, we used data from Marks et al. who compared outcomes for 293 PWID hospitalized with invasive infections who either completed a full course of inpatient IV antibiotics or received oral antibiotics upon patient-directed discharge following a partial course of IV antibiotics.[21] Within this study, 83 PWID initiated oral antibiotics following a patient-directed discharge and 8/83 did not complete a full course of oral antibiotics.[21] Therefore, 75/83 or 90.4% successfully completed a full course of PO following hospitalization. We converted this to an overall rate of failure and scaled the probability by the average duration of PO regimen (i.e., 3 weeks) for 3.3% weekly probability of unlinking from PO treatment.

Cost

Costs included within the model include the cost of medication (antibiotics) as well as treatment utilization, including physician visits and typical laboratory testing.

Pharmaceutical costs

To specify antibiotic regimens and estimate associated costs, we first estimated the distribution of organisms leading to DUA-IE (described fully within the Pharmaceutical costs subsection of the OPAT subsection).

Using this distribution of organisms, we then used recommended regimens reported in Marks et al. and used within the POET trial to assign the most likely oral antibiotic regimen.[21, 22] Due to the unlikelihood of an oral antibiotic regimen being prescribed for a Methicillin-resistant *Staphylococcus aureus* (MRSA)

infection because of a lack of evidence base, we assumed that individuals with DUA-IE related to MRSA would not be eligible for PO and would remain hospitalized for the duration of their antibiotic treatment. This was varied in sensitivity analyses.

We then used medication costs using the Federal Supply Schedule (https://www.va.gov/opal/nac/fss/pharmPrices.asp, accessed 2/23/2021). If the medication was not included within the FSS, we used the average wholesale price and subtracted by 23% to estimate cost. If multiple prices were reported for the same medication, we averaged the price and used the costs to create a range for the probabilistic sensitivity analyses. The weighted weekly cost of outpatient oral antibiotics was $260.71 ($17.29-1,170.03) per week.

Treatment costs

Treatment costs associated with receiving PO at home were derived from Marks et al. and consulting with an expert panel.[21] We assumed that, on average, patients on a PO regimen would receive the following services: a physician visit every two weeks, weekly nurse visit, biweekly complete blood count (CBC) with differential, biweekly liver function testing, and biweekly blood urea nitrogen and creatinine testing. In addition, we assumed that 10% of patients on OPAT would require a CT angiogram and 5% of patients would require an echocardiogram due to suspected septic emboli or other complications based on expert opinion. Costs were calculated in 2020 USD using the Physician Fee Schedule and Laboratory Fee Schedule (both accessed on 4/19/2021). On average, the cost of partial oral antibiotics was $380.56 ($137.14-$1,289.88).

Incremental Cost Effectiveness Ratios (ICERs)

Following guidance from the Second Panel on Cost-Effectiveness in Health and Medicine, ICERs were calculated as the difference in costs between the intervention and comparator (*status quo*) scenario divided by the difference in health benefits.[24] Costs and LYs were discounted at a rate of 3% in line with current recommendations.

eMethods 4. Model Scenarios, Scenario Analyses, Threshold Analyses, and Probabilistic Sensitivity Analyses

Model scenarios

We used the REDUCE model to compare the following treatment strategies for DUA-IE: 1) 4-6 weeks of inpatient IVA along with opioid detoxification, status quo (SQ); 2) 4-6 weeks of inpatient IVA along with inpatient addiction care services (ACS) which offers medications for opioid use disorder (SQ with ACS); 3) 3 weeks of inpatient IVA with ACS followed by OPAT (OPAT); and 4) 3 weeks of IVA with ACS followed by PO antibiotics (PO). Key input parameters are summarized in **eTable 12**.

For this analysis, we simulated a cohort over a lifetime in order to estimate long-term outcomes including: mortality and hospitalizations attributable to DUA-IE, the average percent completing treatment for DUA-IE, life-expectancy, average cost per person, and incremental cost-effectiveness ratios (ICERs). We compared costs using a payer system perspective and denominate currency in 2020 US dollars. We discounted all costs and benefits by 3% annually and expressed ICERs as cost per life-year gained with a willingness-to-pay threshold of $100,000 per LY (19). Probabilistic sensitivity analyses and a threshold analysis were performed to evaluate major findings. Additional details and findings are reported within the accompanying manuscript.

Scenario analyses

Deterministic scenario analyses were performed to evaluate the robustness of the model results to uncertainty in the input parameters. These were run with half a million individuals over a lifetime and compared on key outcomes. We varied the 1) percentage of DUA-IE patients eligible for PO (tied to non-MRSA percentage); 2) the percentage of patients leaving the hospital with patient-directed discharge or against medical advice (from 5% to 2.5% weekly); 3) treatment uptake of OPAT and PO (from 99% to 50% for OPAT and from 79% to 50% for PO); 4) where the rate of overdose within the community and outpatient settings is quadrupled, 5) the uptake of ACS and MOUD while inpatient is increased to 75% from and 6) inpatient stay and average cost of medication within the PO scenario. These results are presented within Table 4 in the accompanying manuscript.

Threshold analyses

We conducted threshold analyses to determine what value for selected parameters (i.e., treatment uptake, treatment completion) changed our major findings. These were run with half a million individuals over a lifetime and compared on key outcomes. Average discounted costs per person, discounted life-years, and ICERs are presented in **eTable 13** while Figure 1 within the accompanying manuscript outlines the impact of these values on our major findings. **eFigure 1** and **eFigure 2** present the threshold values for treatment uptake and costs of OPAT and PO.

Probabilistic sensitivity analyses

We ran probabilistic sensitivity analyses (credible intervals presented in Table 3 within the manuscript) where we held the percent of patients leaving against medical advice and treatment uptake constant while varying the uptake of MOUDs and ACS while hospitalized, cost of antibiotics, and the probability of discontinuing antibiotics post-hospitalization. Parameter distributions presented in Table 2 within the accompanying manuscript.

© 2022 Adams JW et al. *JAMA Network Open*.

eTable 1. Initializing Cohort Characteristics

Parameter	Mean or percentage	Source
Ever injection drug use status	100%	Assumed
Age	41.85 years	Lansky et al.[2], Martins et al.[3], Degenhardt et al.[4], NHBS 2015[5], U.S. Census 2016
Sex	70% male	Lansky et al.[2], Martins et al.[6], Degenhardt et al.[4], NHBS 2015[5], U.S. Census 2016
Injection frequency	53% high frequency<br>11% low frequency<br>36% no current use	Tan et al.[7], Buresh et al.[8]
Reusing/sharing equipment	44.4% reusing/sharing	Stein et al.[9]
Sterile injection practice	65.9% no-cleaning	Stein et al.[9]

© 2022 Adams JW et al. *JAMA Network Open*.

eTable 2. Calibration Targets for Incidence of Endocarditis and Fatal Overdose

Target	Target value	Modeled value	Standard error
1-year endocarditis among PWID	55 per 10,000	55 per 10,000	5.80%
1-year fatal overdose among PWID	68 per 10,000	70 per 10,000	0.74%
3-year endocarditis mortality among PWID	29%	35%	17.4%
Remaining life expectancy among PWID	35.3 years	35.5 years	0.62%

© 2022 Adams JW et al. *JAMA Network Open*.

eTable 3. Weekly Probability of Developing Infective Endocarditis, Stratified by Injection Behavior Profile

Injection behavior profile	Value (mean, standard deviation)
High frequency, higher infectious risk	0.067 (0.0045)
High frequency, lower infectious risk	0.020 (0.0004)
Low frequency, higher infectious risk	0.048 (0.0023)
Low frequency, lower infectious risk	0.014 (0.0002)

© 2022 Adams JW et al. *JAMA Network Open*.

eTable 4. Hospital-Based Services

**Hospital-based service**	**SDU for which service applies (eligibility)**	**Effect in the model**	**Independent cost**
**Addiction consult service**	OD, IE, combination	25.8% of inpatient hospitalizations receive an addiction consult by the end of hospitalization, which increases the probability of linkage to outpatient addiction care and probability of linkage to outpatient MOUD. Changes the probability of transitioning between injection frequency states. See treatment effect description below.	Yes, $225/weekly while inpatient
**Initiation of MOUD (e.g., buprenorphine)**	OD, IE, combination	25% of inpatient hospitalizations receive inpatient MOUDs by the end of hospitalization, which increases the probability of linkage to outpatient MOUD. Changes the probability of transitioning between injection frequency states.	No, included in costs of hospitalization

© 2022 Adams JW et al. *JAMA Network Open*.

eTable 5. Outpatient Addiction Services

**Outpatient service**	**Eligibility**	**Effect in the model, while linked**	**Independent cost**
Outpatient addiction with MOUD	Any ever IDU in the simulation not actively hospitalized	Decreases probability of unclean injection; decreases needle sharing, increases probability of moving to lower frequency state and decreases probability of moving out of no/low frequency state.	Yes
Outpatient addiction without MOUD	Any ever IDU individual in the simulation not actively hospitalized	Decreases probability of unclean injection; decreases needle sharing.	Yes

© 2022 Adams JW et al. *JAMA Network Open*.

eTable 6. Model Parameters Implemented With Outpatient Parenteral Antimicrobial Therapy (OPAT) Module

Parameter	Value, range	Source
Percent uptake	99%, (50-99%)	Expert opinion
Duration of treatment	3 (2-4) inpatient followed by 3 (2-4) outpatient	Fanucchi et al.[15]
Weekly probability of discontinuing outpatient treatment/readmission	4.54% (3-14%)	Fanucchi et al.[15], D'Couto et al.[14], Suzuki et al.[13]

© 2022 Adams JW et al. *JAMA Network Open*.

eTable 7. Estimated Weekly Cost of Medication for Outpatient Intravenous Antibiotic Therapy

**Organism** (Rodger et al.[16], Hartnett et al.[17])	**Percent of DUA-IE infections** (Rodger et al.[16], Hartnett et al.[17])	**Recommended regimen** (American Heart Association[18], expert opinion)	**Weekly cost, mean and range** (FSS)
Methicillin-resistant *Staphylococcus aureus*	21%	Vancomycin 30mg/kg daily in 2 equally divided doses[a]	$100.03 ($74.34-137.19)
Methicillin-sensitive *Staphylococcus aureus*	56%	Cefazolin 6g daily in 3 equally divided doses	$160.41
Streptococci	11%	Aqueous crystalline penicillin G sodium 12–18 million U/24 h IV either continuously or in 4 or 6 equally divided doses[b]	$120.43 ($111.20-126.63)
Enterococci	6%	Ampicillin sodium 2g every four hours OR Aqueous crystalline penicillin G sodium 18-20 million U/24 h IV either continuously or in 6 equally divided doses plus gentamicin sulfate 3mg/kg in 2-3 equally divided doses	$124.80 ($103.03-139.31)
Other (polymicrobial, culture negative, etc.)	6%	Ceftriaxone 2g daily via IV or IM in one dose	$23.87 ($18.29-40.66)
**TOTAL WEIGHTED COST**			**$133.00 ($124.95-143.36)**

[a] Assumed a daily dose of 1,500mg for pricing.
[b] Assumed a daily dose of 15 million units for pricing.

© 2022 Adams JW et al. *JAMA Network Open*.

eTable 8. Estimated Weekly Cost of Treatment Services Related to Outpatient Parenteral Antimicrobial Therapy (OPAT)

Treatment service description	Frequency	Weekly Cost
**While inpatient**		
PICC line insertion (CPT 36569)	One time	$95.29
Chest x-ray (CPT 71020)	One time	$31.17
**TOTAL COST OF OPAT WHILE INPATIENT**		**$126.46**
**Post-discharge from acute care facility**		
MD visit, established patient (Level 3, CPT 99213)	Biweekly	$92.47*0.5= $46.24
Initial specialist nurse visit, weekly nursing visit, weekly supplies	Weekly	$224
Complete blood count with differential (CPT 85025)	Weekly	$7.77
Liver function test (CPT 80076)	Weekly	$8.17
Blood urea nitrogen and creatinine (CPT 80069)	Weekly	$8.68
CT Angiogram chest with and without IV contrast (CPT 71275)	Needed for 10% of patients	$308.46*0.10= $30.85
Echocardiogram	Needed for 5% of patients	$207.96*0.05=$10.40
Antibiotics (from eTable 7)	Weekly	$133.00 ($124.95-143.36)
**Total weekly cost of home-based OPAT**		**$469.10 ($461.05-479.46)**
**Total weekly cost of OPAT at post-acute facility**		**$2,702.00 ($761.94-11,756.36)**
**Weekly cost of OPAT in main analysis (assumes 50% receive at-home OPAT and 50% receive OPAT at a post-acute facility)**		**$1,585.55 ($611.50-6,117.91)**

© 2022 Adams JW et al. *JAMA Network Open*.

eTable 9. Model Parameters Implemented With Partial Oral (PO) Antibiotic Therapy Module

Parameter	Value, range	Source
Percent uptake	79% (50-85%)	Rodger et al.[16]
Duration of treatment	3 weeks	Marks et al.[21]
Weekly probability of discontinuing outpatient treatment	3.3% (2-11%)	Marks et al.[21], expert opinion

© 2022 Adams JW et al. *JAMA Network Open*.

eTable 10. Estimated Weekly Cost of Medication for Outpatient Oral Antibiotic Therapy

**Organism** (Rodger et al.[16], Hartnett et al.[17])	**Percent of DUA-IE infections**[16, 17]	**Recommended regimen** (Marks et al.[23], Iversen et al.[22], expert opinion)	**Weekly cost, mean and range** (FSS, AWP)
Methicillin-resistant *Staphylococcus aureus*	21%	Not eligible for PO, standard hospitalization	n/a
Methicillin-sensitive *Staphylococcus aureus*	56%	Sulfamethoxazole 800mg/Trimethoprim 160mg 2 tablets twice daily **OR** Linezolid 600mg tablets 1 tablet twice daily **OR** Doxycycline 100 mg twice daily + Sulfamethoxazole 800mg/Trimethoprim 160mg 2 tablets twice daily[a]	$288.15 ($11.93-1,334.11)
Streptococci	11%	Amoxicillin 875mg/clavulanate 125mg 1 tablet twice daily + Levofloxacin 500 mg 1 tablet daily	$50.11 ($31.96-99.66)
Enterococci	6%	Linezolid 600mg tablets 1 tablet twice daily + Rifampin 300 mg 2 tablets twice daily **OR** Amoxicillin 875mg/clavulanate 125mg 1 tablet twice daily + Levofloxacin 500 mg 1 tablet daily[b]	$406.09 ($39.15-1,795.12)
Other (polymicrobial, culture negative, etc.)	6%	Linezolid 600mg tablets 1 tablet twice daily + Rifampin 300 mg 2 tablets twice daily **OR** Levofloxacin 500 mg 1 tablet daily **OR** Ciprofloxacin 750mg twice daily + Doxycycline 100 mg twice daily[c]	$247.50 ($18.37-1,003.62)
**TOTAL**			**$260.71 ($17.29-1,170.03)**

[a] Assumes each regimen is used by 33.3% of patients

[b] Assumes each regimen is used by 50% of patients

[c] Assumes 21% of patients used Linezolid+ rifampin, 39.5% of patients used levofloxacin, and 39.5% of patients used Ciprofloxacin+Doxycycline

© 2022 Adams JW et al. *JAMA Network Open*.

eTable 11. Estimated Weekly Cost of Treatment Services Related to Partial Oral (PO) Antibiotic Therapy

Treatment strategy	Percent uptake[a]	Average duration of treatment (weeks, range)	Percent successfully completing treatment	Weekly cost per treated infection[b]	Sources
**Status quo (inpatient for duration of treatment)**	100%	6 (4-8) inpatient	70%	$21,573.19 (SD: $12,837.01)	Miller et al.(4),
**Outpatient parenteral antimicrobial therapy (OPAT)**	99% (assumed), only ~50% eligible for home-based OPAT	3 (2-4) inpatient, followed by 3 (2-4) weeks outpatient	87%	$1,585.55 (range: $611.50-6,117.91)	Eaton et al. (22), Suzuki et al. (8), D'Couto et al. (7), Beieler et al. (23), Fanucchi et al. (6)
**Partial oral (PO) antibiotics**	79% (only non-MRSA infections eligible)	3 (2-4) inpatient, followed by 3 (2-4) weeks outpatient	90.4%	$380.56 (range: $137.14-1,289.88)	Rodger et al.(24) Marks et al.(14),

© 2022 Adams JW et al. *JAMA Network Open*.

eTable 12. Key Input Parameters for Treatment Strategies Modeled Within REDUCE to Compare Approaches to Treating Drug Use–Associated Endocarditis

**Treatment service description**	**Frequency**	**Weekly Cost**
MD visit, established patient (Level 3, CPT 99213)	Biweekly	$92.47*0.5= $46.24
Weekly nursing visit (CPT 99211)	Weekly	$20.06
Complete blood count with differential (CPT 85025)	Biweekly	$3.89
Liver function test (CPT 80076)	Biweekly	$4.09
Blood urea nitrogen and creatinine (CPT 80069)	Biweekly	$4.34
CT Angiogram chest with and without IV contrast (CPT 71275)	Needed for 10% of patients	$308.46*0.10= $30.85
Echocardiogram	Needed for 5% of patients	$207.96*0.05=$10.40
Antibiotics (from eTable 10)	Weekly	$260.71 ($17.29-1,170.03)
**TOTAL WEEKLY COST OF PO**		**$380.56 ($137.14-$1,289.88)**

Abbreviations: SD- standard deviation

[a] Number of patients initiating treatment regimen divided by the total number offered.

[b] Includes hospitalization costs, medication costs, and health worker time.

© 2022 Adams JW et al. *JAMA Network Open*.

eTable 13. Cost-Effectiveness Outcomes From Threshold Analyses

Model scenario	Parameter varied	Value in main analysis	Threshold value	Avg discounted life year	Average discounted cost	ICER
1 (OPAT)	Treatment uptake	99%	79%	18.655	$413,860	n/a
2 (PO)	Treatment uptake	79%	86%	18.670	$ 413,749	$72,182
3 (OPAT)	Treatment discontinuation	4.54%/wk	6.01%/wk	18.640	$412,307	n/a
4 (PO)	Treatment discontinuation	3.3%/wk	2.65%/wk	18.670	$414,203	$99,065
5 (PO)	Treatment discontinuation	3.3%/wk	7.3%/wk	18.636	$413,546	n/a
6 (OPAT)	Weekly cost of treatment	$1,711	$21,000	18.651	$416,875	$0
7 (OPAT)	Weekly cost of treatment	$1,711	$27,000	18.651	$418,141	$96,135
8 (PO)	Weekly cost of treatment	$381	$0	18.663	$414,010	$118,962

© 2022 Adams JW et al. *JAMA Network Open*.

eFigure 1. Threshold Values for Treatment Uptake of Partial Oral (PO) Antibiotic Therapy and Outpatient Parenteral Antimicrobial Therapy (OPAT)

Treatment Uptake

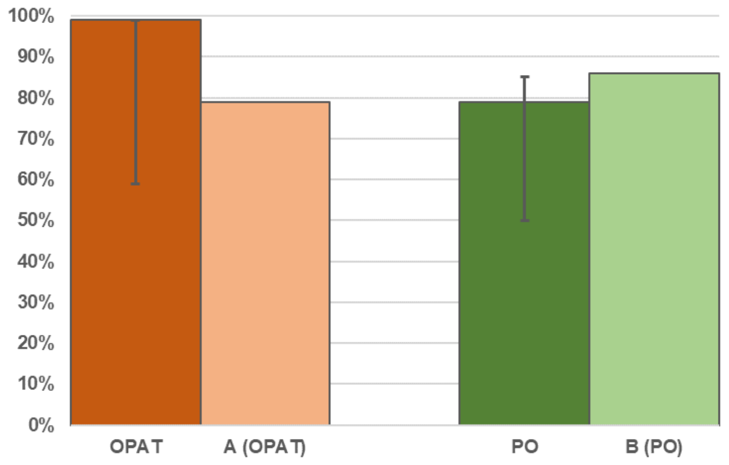

Legend

Model scenario	Parameter change	Effect in model
A (OPAT)	Treatment uptake decreased from 99% to 79%	No longer less expensive compared to PO (dominated)
B (PO)	Treatment uptake increased from 79% to 86%	Cost-effective compared to OPAT

[a] Error bars present upper and lower range of the uniform distribution implemented in the probability sensitivity analyses, unless indicated otherwise.
* Status quo scenarios implemented a normal distribution with standard deviation (one standard deviation range shown here).

© 2022 Adams JW et al. *JAMA Network Open*.

eFigure 2. Threshold Values for Weekly Cost of Treatment for Partial Oral (PO) Antibiotic Therapy and Outpatient Parenteral Antimicrobial Therapy (OPAT)

Weekly Cost of Treatment

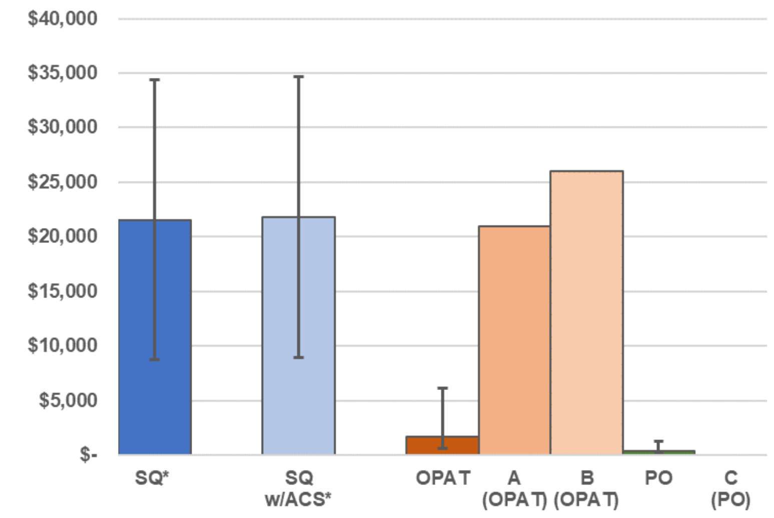

Legend

Model scenario	Parameter change	Effect in model
A (OPAT)	Weekly cost of treatment increased from $1,711 to $21,000	No longer cost-saving compared to SQ
B (OPAT)	Weekly cost of treatment increased from $1,711 to $26,000	No longer cost-effective compared to SQ at $100,000 threshold
C (PO)	Weekly cost of treatment decreased from $381 to $0	Even at no cost, PO not cost-effective compared to OPAT due to proportion hospitalized

[a] Error bars present upper and lower range of the uniform distribution implemented in the probability sensitivity analyses, unless indicated otherwise.
* Status quo scenarios implemented a normal distribution with standard deviation (one standard deviation range shown here).

© 2022 Adams JW et al. *JAMA Network Open*.

eReferences

1. Barocas JA, Eftekhari Yazdi G, Savinkina A, et al. Long-term Infective Endocarditis Mortality Associated With Injection Opioid Use in the United States: A Modeling Study. *Clinical Infectious Diseases*. 2020;doi:10.1093/cid/ciaa1346
2. Lansky A, Finlayson T, Johnson C, et al. Estimating the Number of Persons Who Inject Drugs in the United States by Meta-Analysis to Calculate National Rates of HIV and Hepatitis C Virus Infections. *PLOS ONE*. 2014;9(5):e97596. doi:10.1371/journal.pone.0097596
3. Martins SS, Sarvet A, Santaella-Tenorio J, Saha T, Grant BF, Hasin DS. Changes in US Lifetime Heroin Use and Heroin Use Disorder: Prevalence From the 2001-2002 to 2012-2013 National Epidemiologic Survey on Alcohol and Related Conditions. *JAMA Psychiatry*. 2017;74(5):445-455. doi:10.1001/jamapsychiatry.2017.0113
4. Degenhardt L, Peacock A, Colledge S, et al. Global prevalence of injecting drug use and sociodemographic characteristics and prevalence of HIV, HBV, and HCV in people who inject drugs: a multistage systematic review. *The Lancet Global Health*. 2017/12/01/ 2017;5(12):e1192-e1207. doi:https://doi.org/10.1016/S2214-109X(17)30375-3
5. Adams ML, Wejnert C, Finlayson T, Xia M, Paz-Bailey G. HIV Infection, risk, prevention, and testing behaviors among persons who inject drugs: National HIV Behavioral Surveillance: injection drug use, 20 US cities, 2015. 2017;
6. Banerjee G, Edelman EJ, Barry DT, et al. Non-medical use of prescription opioids is associated with heroin initiation among US veterans: a prospective cohort study. *Addiction*. 2016/11/01 2016;111(11):2021-2031. doi:10.1111/add.13491
7. Tan S, Makela S, Heller D, et al. A Bayesian evidence synthesis approach to estimate disease prevalence in hard-to-reach populations: hepatitis C in New York City. *Epidemics*. 2018/06/01/ 2018;23:96-109. doi:https://doi.org/10.1016/j.epidem.2018.01.002
8. Buresh M, Genberg BL, Astemborski J, Kirk GD, Mehta SH. Recent fentanyl use among people who inject drugs: Results from a rapid assessment in Baltimore, Maryland. *International Journal of Drug Policy*. 2019/12/01/ 2019;74:41-46. doi:https://doi.org/10.1016/j.drugpo.2019.08.006
9. Stein MD, Phillips KT, Herman DS, et al. Skin-cleaning among hospitalized people who inject drugs: a randomized controlled trial. *Addiction*. 2021;116(5):1122-1130. doi:https://doi.org/10.1111/add.15236
10. Kimmel SD, Kim J-H, Kalesan B, Samet JH, Walley AY, Larochelle MR. Against medical advice discharges in injection and non-injection drug use-associated infective endocarditis: A nationwide cohort study. *Clinical Infectious Diseases*. 2020;doi:10.1093/cid/ciaa1126
11. Meisner JA, Anesi J, Chen X, Grande D. Changes in Infective Endocarditis Admissions in Pennsylvania During the Opioid Epidemic. *Clinical Infectious Diseases*. 2020;71(7):1664-1670. doi:10.1093/cid/ciz1038
12. Mitchell ED, Czoski Murray C, Meads D, Minton J, Wright J, Twiddy M. Clinical and cost-effectiveness, safety and acceptability of community intravenous antibiotic service models: CIVAS systematic review. *BMJ Open*. 2017;7(4):e013560. doi:10.1136/bmjopen-2016-013560
13. Suzuki J, Johnson J, Montgomery M, Hayden M, Price C. Outpatient Parenteral Antimicrobial Therapy Among People Who Inject Drugs: A Review of the Literature. *Open forum infectious diseases*. 2018;5(9):ofy194-ofy194. doi:10.1093/ofid/ofy194
14. D'Couto HT, Robbins GK, Ard KL, Wakeman SE, Alves J, Nelson SB. Outcomes According to Discharge Location for Persons Who Inject Drugs Receiving Outpatient

Parenteral Antimicrobial Therapy. *Open Forum Infectious Diseases*. 2018;5(5)doi:10.1093/ofid/ofy056
15. Fanucchi LC, Walsh SL, Thornton AC, Nuzzo PA, Lofwall MR. Outpatient Parenteral Antimicrobial Therapy Plus Buprenorphine for Opioid Use Disorder and Severe Injection-related Infections. *Clinical Infectious Diseases*. 2019;70(6):1226-1229. doi:10.1093/cid/ciz654
16. Rodger L, Glockler-Lauf SD, Shojaei E, et al. Clinical Characteristics and Factors Associated With Mortality in First-Episode Infective Endocarditis Among Persons Who Inject Drugs. *JAMA Network Open*. 2018;1(7):e185220-e185220. doi:10.1001/jamanetworkopen.2018.5220
17. Hartnett KP, Jackson KA, Felsen C, et al. Bacterial and fungal infections in persons who inject drugs—Western New York, 2017. *Morbidity and Mortality Weekly Report*. 2019;68(26):583.
18. Baddour LM, Wilson WR, Bayer AS, et al. Infective endocarditis in adults: diagnosis, antimicrobial therapy, and management of complications: a scientific statement for healthcare professionals from the American Heart Association. *Circulation*. 2015;132(15):1435-1486.
19. Krah NM, Bardsley T, Nelson R, et al. Economic burden of home antimicrobial therapy: OPAT versus oral therapy. *Hospital pediatrics*. 2019;9(4):234-240.
20. Conant MM, Erdman SM, Osterholzer D. Mandatory infectious diseases approval of outpatient parenteral antimicrobial therapy (OPAT): clinical and economic outcomes of averted cases. *Journal of Antimicrobial Chemotherapy*. 2014;69(6):1695-1700.
21. Marks LR, Liang SY, Muthulingam D, et al. Evaluation of partial oral antibiotic treatment for persons who inject drugs and are hospitalized with invasive infections. *Clin Infect Dis*. Apr 2 2020;doi:10.1093/cid/ciaa365
22. Iversen K, Ihlemann N, Gill SU, et al. Partial Oral versus Intravenous Antibiotic Treatment of Endocarditis. *New England Journal of Medicine*. 2019/01/31 2018;380(5):415-424. doi:10.1056/NEJMoa1808312
23. Marks LR, Liang SY, Muthulingam D, et al. Evaluation of Partial Oral Antibiotic Treatment for Persons Who Inject Drugs and Are Hospitalized With Invasive Infections. *Clinical Infectious Diseases*. 2020;doi:10.1093/cid/ciaa365
24. Neumann PJ, Sanders GD, Russell LB, Siegel JE, Ganiats TG. *Cost effectiveness in health and medicine*. Second edition. ed. Oxford University Press; 2017:xxxiii, 496 pages.

© 2022 Adams JW et al. *JAMA Network Open*.
